# GWAS of grain color and tannin content in Chinese *sorghum* based on whole-genome sequencing

**DOI:** 10.1007/s00122-023-04307-z

**Published:** 2023-03-23

**Authors:** Liyi Zhang, Jianxia Xu, Yanqing Ding, Ning Cao, Xu Gao, Zhou Feng, Kuiying Li, Bing Cheng, Lengbo Zhou, Mingjian Ren, Yuezhi Tao, Guihua Zou

**Affiliations:** 1 Guizhou Institute of Upland Crops, Guizhou Academy of Agricultural Sciences, Guiyang, 550006 China; 2 Institute of Crop and Nuclear Technology Utilization, Zhejiang Academy of Agricultural Sciences, Zhejiang Key Laboratory of Digital Dry Land Crops, Hangzhou, 310021 China; 3 College of Agriculture, Guizhou University, Guiyang, 550025 China

## Abstract

**Key message:**

Seventy-three QTL related to grain color and tannin content were identified in Chinese *sorghum* accessions, and a new recessive allelic variant of *TAN2* gene was discovered.

**Abstract:**

*Sorghum* is mainly used for brewing distilled liquors in China. Since grain tannins play an important role in liquor brewing, accurately understanding the relationship between grain color and tannin content can provide basis for selection standards of tannin *sorghum*. We resequenced a panel of 242 Chinese *sorghum* accessions and performed population structure and genome-wide association study (GWAS) to identify quantitative trait locus (QTL) affecting pericarp color, testa pigment, and tannin content. Phylogenetic analysis, principal component analysis (PCA), and admixture model were used to infer population structure. Two distinct genetic sub-populations were identified according to their corresponding northern and southern geographic origin. To investigate the genetic basis of natural variation in *sorghum* grain color, GWAS with 2,760,264 SNPs was conducted in four environments using multiple models (Blink, FarmCPU, GLM, and MLM). Seventy-three QTL were identified to be associated for the color of exocarp, mesocarp, testa, and tannin content on all chromosomes except chromosome 5, of which 47 might be novel QTL. Some important QTL were found to colocalize with orthologous genes in the flavonoid biosynthetic pathway from other plants, including orthologous of Arabidopsis (*Arabidopsis thaliana*) *TT2*, *TT7*, *TT12*, *TT16* and *AT5G41220* (*GST*), as well as orthologous of rice (*Oryza sativa*) *MYB61* and *OsbHLH025*. Our investigation of the variation in grain color and tannin content in Chinese *sorghum* germplasm may help guide future *sorghum* breeding for liquor brewing.

**Supplementary Information:**

The online version contains supplementary material available at 10.1007/s00122-023-04307-z.

## Introduction

*Sorghum* (*Sorghum bicolor* (L.) Moench, 2*n* = 2*x* = 20) is the world's fifth crop and has a wide range of applications as food, feed, fiber, and bio-energy feedstocks. In China, *sorghum* has been used as critical ingredient for brewing liquors ever since Yuan Dynasty (1270–1368 CE) (Shinoda 1958). At present, *sorghum* is mainly cultivated in the Southwest, North, and Northeast regions of China and more than 80% of *sorghum* grain are used for liquor-making (Chen et al. 2019). Almost all Chinese famous brands of distilled liquors, such as Moutaijiu (Moutai-aroma liquor), Luzhoulaojiao, Wuliangye (strong-aroma liquor), and Fenjiu (light-aroma liquor), are brewed with *sorghum* grain as a key ingredient. Among biochemical and physical characteristics related liquor-making quality, tannins (proanthocyanidins, PAs) in the *sorghum* grains play a significant role in brewing Chinese liquor because tannins not only inhibit the growth of miscellaneous bacteria but also produce syringic acid and syringaldehyde essential for the unique flavor during the brewing process (Yang et al. 2020). Moreover, *sorghum* grains with distinct tannin content are needed for brewing liquors with different aroma, taste, and flavor. Thus, it is very important to breed elite *sorghum* varieties with suitable tannin content to meet the needs of liquor brewing industry for producing variety of liquors with distinct aroma.

In general, the concentration of tannin in *sorghum* cultivars is related to the grain color, for example, red and brown grains usually contain more tannins (Kumari et al. 2021). Breeders throughout history rely primarily on grain color, instead of tannin content, for breeding high tannin *sorghum* through conventional breeding. In fact, pericarp color is not a reliable indicator of *sorghum* tannin contents. Except for condensed tannins, *sorghum* grains have a wide array of phenolic compounds in the pericarp including phenolic acids and flavonoids, which are responsible for various coloration in the grains. The classical genetic models reveal that several loci affect grain color and testa pigment (Dykes 2019). The base pericarp color is controlled by *R* and *Y* genes, and can appear as red, yellow, or white, corresponding to the genotypes with *R_Y_*, *rrY_*, and *R_yy* (or *rryy*), respectively. Pericarp color does not affect the presence of a pigmented testa or the presence of tannins (Dykes et al. 2013). *B1* and *B2* loci control tannin synthesis in grain testa. Due to the presence of a spreader (*S*) gene, genotypes with *B1_B2_ ss* have tannins in the testa, while genotypes with *B1_B2_S_* have tannins in both pericarp and testa, usually resulting in grains with brown pericarp. In addition, several other genes can modify the base pericarp color, such as intensifier (*I*) and mesocarp thickness (*Z*), resulting in a range of colors from brilliant white to black in *sorghum* genotypes (Earp et al. 2004b).

Tannins belong to flavonoids which are secondary metabolites in higher plants known for the pigmentation in flowers, fruits, and seeds (Petroni and Tonelli 2011). Among the major cereal crops, *sorghum* grains are rich in condensed tannins, which affect seed dormancy (Debeaujon et al. 2000), protect against grain mold (Nida et al. 2021b) and bird and insect predation (Wu et al. 2019; Xie et al. 2019). Anthocyanins and proanthocyanidins are produced by a specific branch of the flavonoid pathway, involving in dozens of structural genes and multiple regulatory genes, which have been identified and are well characterized in maize and *Arabidopsis* (Chatham et al. 2019; Petroni and Tonelli 2011). To date, only three regulatory genes are identified in *sorghum*. *Tannin1* (*TAN1*, Sobic.004G280800) encodes a WD40 protein and is a gene underlying *B2* locus (Wu et al. 2012), which is a orthologue to *TRANSPARENT TESTA GLABRA 1* (*TTG1*), a regulator of proanthocyanidins in Arabidopsis. *Tannin2* (*TAN2*, Sobic.002G076600) encodes a bHLH domain protein and is a gene underlying *B1* locus, which is the orthologue of *TT8* (Arabidopsis), *Rc* (rice) and *IN1* (maize) genes involved in the phenylpropanoid biosynthetic pathway (Wu et al. 2019). Furthermore, a MYB transcription factor, *Yellow seed1* (*Y1,* Sobic.001G398100), which controls pericarp pigmentation and 3-deoxyanthocyanidins accumulation in *sorghum* pericarp, has been cloned (Ibraheem et al. 2010; Nida et al. 2019; Tao et al. 2021a). Therefore, huge gaps remain in understanding the underlying genetic mechanisms of the diversity of tannin and grain color in *sorghum* compared to other crops.

In this study, we took advantage of the whole-genome resequenced 242 diverse Chinese *sorghum* lines to further explore intrinsic relationship between grain color and tannin content and to detect novel QTL for the two traits. Population structure analysis was conducted to investigate genetic diversities of Chinese *sorghum*. GWAS was performed to detect QTL for the color of pericarp and testa, as well as tannin contents. Our study identified several important candidate genes for tannin biosynthesis and demonstrated that breeding tannin varieties based on grain color alone is likely to lead to relatively high deviations in grain tannin content. Our results also provide a theoretical basis for breeding *sorghum* for brewing liquor using marker-assisted selection (MAS).

## Materials and methods

### Plant material and phenotyping

A total of 242 Chinese *sorghum* accessions from the Center for Crop Germplasm Resources, Institute of Crop Sciences, Chinese Academy of Agricultural Science (Beijing, China), and Institute of Upland Crops, Guizhou Academy of Agricultural Science (Guiyang, China) were used for this study (Supplementary Table 1).

**Fig. 1:**
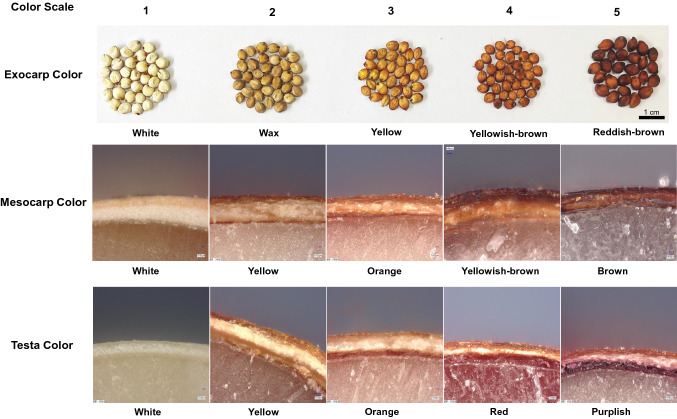
Schematic diagram of the five-scoring level for the color of exocarp (EC), mesocarp (MC) and testa (TC) in *sorghum* grain. EC was observed with the naked eye, and the scale bar is 1 cm. MC and TC were viewed under a Digital Microscope at 500X magnification, and the scale bar is 10 μm (color figure online)

From 2018 to 2020, the panel of 242 accessions were grown in Guiyang [26.67° N, 106.62° E], Guizhou Province; and Ledong [18.74° N, 109.17° E], Hainan Province. All experiments were planted in a randomized block design with two replications. Each plot contained two rows of 15 plants each, with spacing of 40 cm between rows and 20 cm within rows. Each row was over planted and later thinned to 1 plant per hole after 3–4 weeks. The fields were irrigated and fertilized following standard local cultivation practices. In brief, 100 kg ha^−1^ composite fertilizer was applied to the experimental plots at sowing. To avoid cross-pollination, all plants were bagged before flowering. Three main stem panicles from each plot were harvested 6 weeks after heading and sun-dried for two weeks. Seeds were threshed and stored in dry containers at room temperature. Grain exocarp color (EC) was evaluated and scored as five levels: 1 (White), 2 (Wax), 3 (Yellow), 4 (Yellowish-brown), and 5 (Reddish-brown), grain mesocarp color (MC) and testa color (TC) were evaluated and scored as five levels: 1 (white), 2 (yellow), 3 (orange), 4 (Yellowish-brown/red), and 5 (Brown/purplish) by using a Digital Microscope (VHX-7000, KEYENCE Technology Company, Shanghai, China) (Fig. 1).

Tannin content was quantified through using Chinese national standard GB/T 15,686-2008. One gram of grains from each genotype was ground and added 20 ml of dimethylamide. After mixing and centrifugation, two tubes of 1 ml supernatant were added ammonia solution and ammonia solution-ferric ammonium citrate, respectively. After mixing and resting for 10 min, the absorbance was measured at 525 nm with a spectrophotometer, and the difference between the two absorbance values was the concentration of tannic acid (mg/mL).

### DNA extraction and sequencing

Genomic DNA was extracted following the cetyltrimethylammonium bromide (CTAB) method and quantified by Qubit® 2.0 fluorescent meter (Invitrogen, Carlsbad, USA). The quality of the DNA was determined by electrophoresis in 0.8% agarose running at 100 V for 40 min. The DNA fragments around 350 bp were randomly generated by Covaris ultrasonic crushing apparatus. The constructed library was used for paired-end (PE150) sequencing on Illumina HiSeq 4000 sequencing platform by Beijing Novogene technology co., LTD (Beijing, China).

### Reads mapping and variants calling

The raw paired-end reads were trimmed and filtered with a sliding window of size 4, with average Phred score scale of 20 within the window using fastp (version: 0.20.0) (Chen et al. 2018). The clean reads of 242 accessions were mapped to the *Sorghum* bicolor genome (Phytozome v13, https://genome.jgi.doe.gov/portal/pages/dynamicOrganismDownload.sf?organism=Phytozome) (Goodstein et al. 2012) using BWA (version: 0.7-17) with default parameters (Li and Durbin 2009). After alignment, Picard tools (version: 2.18.17, http://broadinstitute.github.io/picard/) were used to remove PCR duplicates according to the mapping coordinates.

The variation detection followed the best practice workflow recommended by Genome Analysis Toolkit (GATK4. version 3.8.1) (McKenna et al. 2010). SNPs and InDels were annotated according to the BTx623 genome using the SnpEff (version: 4.3 T) (Cingolani et al. 2012). SNP and InDel density across each chromosome were counted with 500 kb sliding window using VCFtools (version: 0.1.17) software (Danecek et al. 2011).

### Phylogenetic and population analyses

A phylogenetic tree was constructed using the neighbor-joining method in the program PHYLIP (version: 3.697, http://evolution.genetics.washington.edu/phylip. html), and IBS distance matrices were calculated using PLINK. The resulting phylogenetic tree was visualized using the online tool iTOL (version:2.41) (Letunic and Bork 2019). Principal Components Analysis (PCA) was performed with the smartPCA program from EIGENSOFT package (version: 6.1.4) (Price et al. 2006), and the first three eigenvectors were plotted by scatterplot3d R package. Population structure was inferred using the ffastSTRUCTURE (version: 1.0) program (Raj et al. 2014). To explore the convergence of individuals, we predefined the number of genetic clusters K from 2 to 12 and ran the cross-validation error (CV) procedure.

### Genome-wide association study

In total, 2,760,264 high-quality SNPs were obtained after filtration based on MAF > 0.05. All 242 *sorghum* accessions with missing data < 20% were used for GWAS and linkage disequilibrium (LD) analysis. The GWAS was performed with the GLM, MLM, FarmCPU and BLINK statistical methods implemented in Genome Association and Prediction Integrated Tool package (GAPIT version: 3.0) (Lipka et al. 2012). The first three PC derived from whole-genome SNPs were used as fixed effects in the mixed model to correct for stratification. The random effect was estimated from the groups clustered based on the kinship among all accessions. We defined the whole-genome significant cutoff with the adjusted Bonferroni test threshold, which was set as *P* = − log10 (0.05/2,760,264) = 7.6. Based on LD decay distance of 62.5 kb on average (*r*^2^ = 0.2, Supplementary Fig. 1) for the 242 Chinese accessions, QTL was defined as follows: all significant SNPs within a 125 kb were clustered in one interval, where the SNP with smallest *P* values was selected as QTL peak position, and its 62.5 kb upstream and downstream were determined as the QTL confidence interval (Astorkia et al. 2020; Cui et al. 2022; Hu et al. 2019; Tao et al. 2020, 2021b; Wang et al. 2022), which was named as follows: QTL + chromosome + physical location (bp), for example, qtl1.64009126 referred to QTL at position of 64,009,126 bp on chromosome 1. Candidate genes in or near QTL were searched in the BTx623 reference genome, and their putative functions were annotated in the available databases: Phytozome (https://phytozome-next.jgi.doe.gov/), Rice (https://www.ricedata.cn/gene/), Arabidopsis (https://www.arabidopsis.org/), and MaizeGDB (https://www.maizegdb.org/).

### Haplotype analysis of *TAN2* and *Y1* genes

The haplotype analysis of the *TAN2* (Sobic.002G076600) and *Y1* (Sobic.001G398100) gene among the 242 Chinese *sorghum* accessions were performed using the SNPs data on genomic sequence by CandiHap package (version: 0.018). The resulting haplotypes for the gene were visualized using the RectChr package (version: 1.30) (https://github.com/BGI-shenzhen/RectChr).

## Result

### Genome resequencing and population structure

To investigate the population structure of Chinese *sorghum* accessions, we firstly carried out resequencing 242 *sorghum* landraces/cultivars, which were collected extensively from 16 *sorghum* planting provinces across China (151 from North China, 91 from South China) (Supplementary Table 1). Whole-genome resequencing generated a total of ∼8.87 G paired-end reads 150 bp in length, with an average sequencing depth of ∼7.43 and an average genome coverage of ∼87.57%. After mapping to the *sorghum* reference genome BTx623 and single nucleotide polymorphism (SNP) calling, we obtained 2,760,264 high-quality SNPs and 396,358 InDels (insertions-deletions) from the 242 *sorghum* accessions.

**Fig. 2:**
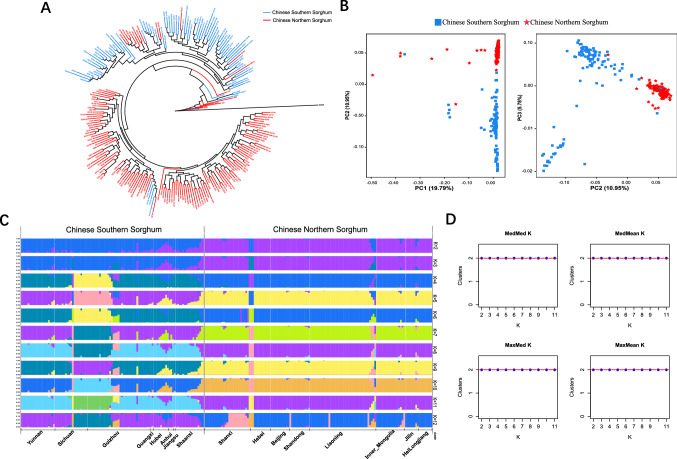
Population structure based on 2,760,264 SNP for 242 Chinese *sorghum*. **A** Neighbor-joining tree for *sorghum* accessions. Red and blue indicate Chinese North and South subgroup, respectively. **B** Principal component analysis of Chinese *sorghum* accessions, showing the first three principal components. Red and blue indicate Chinese North and South subgroup, respectively. **C** Genetic structure as inferred with *fast*structure for *K* = 2 to 12. **D** The optimal *K* value was equal to 2 (*K* = 2) determined by Cross-Validation error (color figure online)

To comprehensively study the relationship between these accessions, we used three methods to infer their population structure. The SNP-based phylogenetic tree divides all accessions into southern and northern groups according to their geographical origins (Fig. 2A). Southern and northern *sorghum* formed four and six subgroups, respectively. However, all the Shaanxi landraces from northern China were clustered into the southern group, mainly forming a clade. Similarly, PCA (principal component analysis) result was consistent with phylogenetic tree analysis (Fig. 2B). The *sorghum* accessions were mainly divided into two genetic groups based on their origin. Approximately 30% of genetic variance was explained by PC1 and PC2, which mainly distinguished southern and northern accessions. PC3 mainly differentiated southwestern accession from Southern accessions of China. Furthermore, admixture model in ffastSTRUCTURE was used to infer population structure of Chinese *sorghum* (Fig. 2C), and the optimal *K* value was equal to 2 (*K* = 2) determined by Cross-Validation error (Fig. 2D). We also found three main clusters were assigned when *K* value from 4 to 11, the accessions from Chinese southwest was differentiated from southern *sorghum* and formed the third group, except for northern and southern groups. Like the results of phylogenetic tree analysis, almost all accessions from Shaanxi province in northern China were clustered in southern *sorghum*. In addition, a new cluster was differentiated from *sorghum* of Shanxi province in northern China at *K* = 12.

## Phenotypic variation in grain color and tannin content

**Fig. 3:**
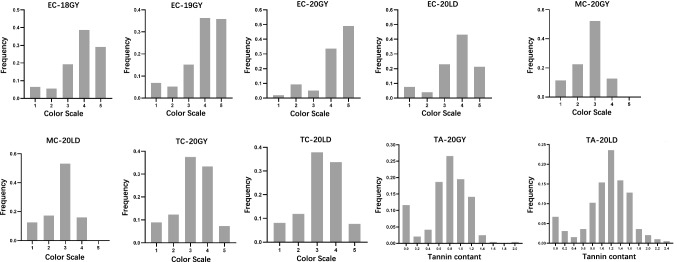
Frequency distribution of exocarp color (EC), mesocarp color (MC), testa color (TC), and tannin content (TA) in environments of Guiyang in 2018 (18GY), 2019 (19GY), and 2020 (20GY), as well as Ledong in 2020 (20LD). Color scale for EC is: 1 (white), 2 (wax), 3 (yellow), 4 (yellowish-brown), and 5 (reddish-brown). Color scale of MC and TC are: 1 (white), 2 (yellow), 3 (orange), 4 (yellowish-brown / Red), and 5 (brown / purplish) (color figure online)

Phenotypic variation for color of pericarp and testa and tannin content were determined in the diverse association panel of 242 accessions from China. From 2018 to 2020, the colors for grain exocarp, mesocarp and testa, as well as tannin content were evaluated in two to four environments (Fig. 3). In general, most Chinese *sorghum* grains were deep in color (color scale from 3 to 5), and 88%, 70% and 80% of accessions presented darker color for exocarp, mesocarp, and testa. The mean values of tannin content were 0.76 ± 0.37 and 1.10 ± 0.49 for grain harvested from Guiyang and Ledong, respectively, indicating that the tannin content of *sorghum* grown in Guiyang was lower than that in Ledong.

**Fig. 4:**
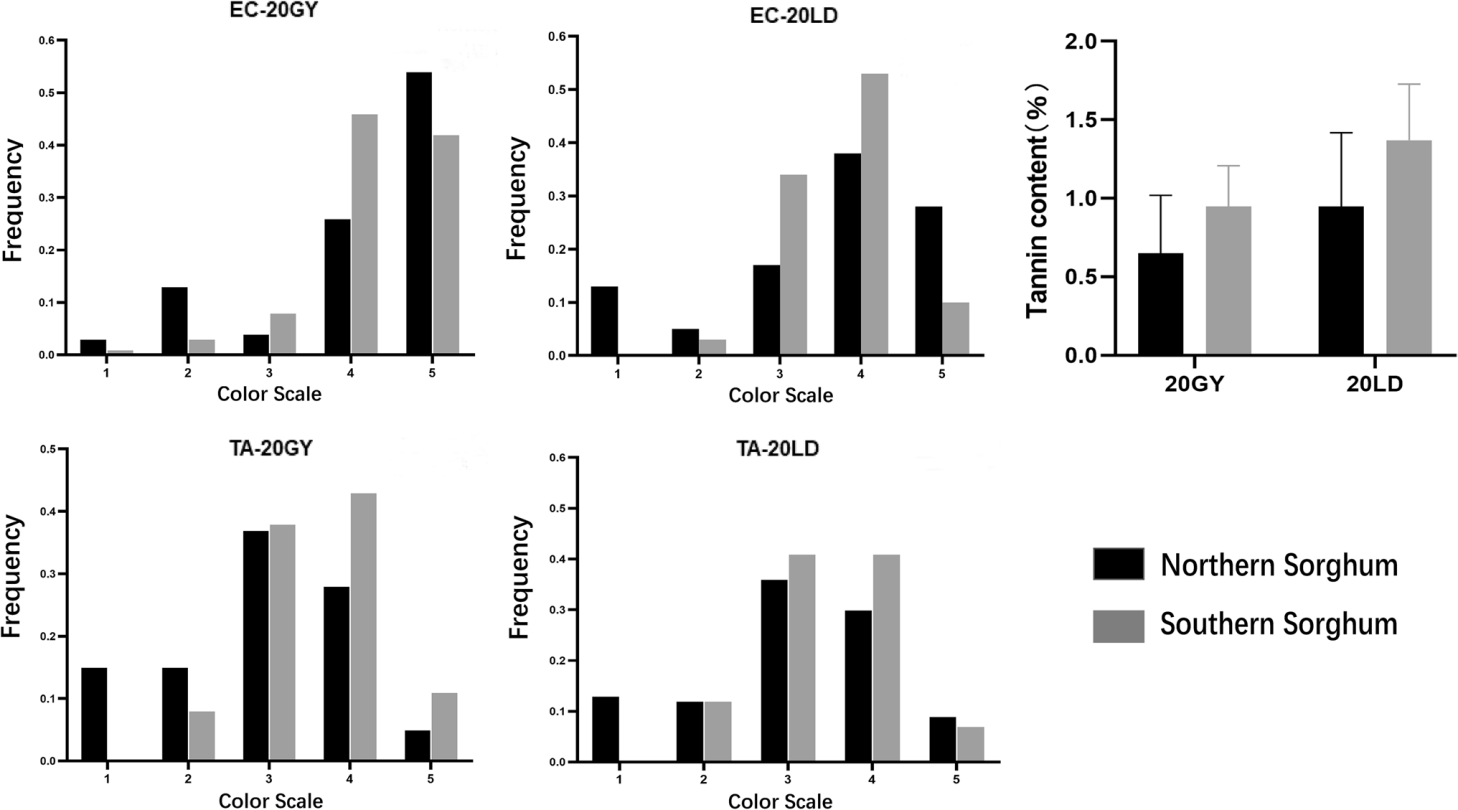
Distribution of exocarp color, testa color and tannin content for Northern and Southern *sorghum* in Guiyang (20GY) and Ledong (20LD) environments in 2020. Color scale for EC is: 1 (white), 2 (wax), 3 (yellow), 4 (yellowish-brown), and 5 (reddish-brown). Color scale of MC and TC are: 1 (white), 2 (yellow), 3 (orange), 4 (yellowish-brown / red), and 5 (brown / purplish). The error bars represent standard deviation (color figure online)

The color distributions of pericarp, testa and tannin content were different between northern and southern *sorghum* (Fig. 4). The frequency of northern *sorghum* with the lightest (Scale 1) and darkest (Scale 5) exocarp color was higher than that of southern *sorghum*, while the exocarp color of southern *sorghum* was more concentrated in the intermediate color scale (3 and 4). A similar distribution was also observed in testa color. However, the frequency of northern *sorghum* with the lightest (Scale 1) mesocarp color was higher than that of southern *sorghum*, while the frequency of northern *sorghum* with darkest (Scale 5) mesocarp color was lower than that of southern *sorghum*. As expected, southern *sorghum* had higher tannin content than northern *sorghum*.

**Fig. 5:**
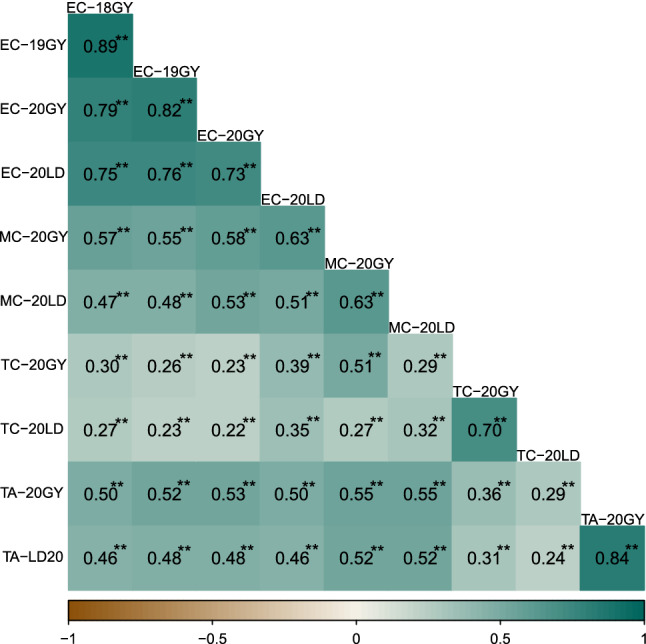
Correlation analysis of exocarp color (EC), mesocarp color (EC), testa color (TC), and tannin content (TA) in different environments. *Indicate significant level less than 0.05; **Indicate significant level less than 0.01 (color figure online)

To investigate the relationship between traits and between environments, we conducted a correlation analysis (Fig. 5). The same traits between different environments showed a high association, with R value from 0.63 to 0.89 at a significant level (*P* < 0.01). The Pearson‘s correlation coefficients between tannin content (0.84) were higher than those between the other three traits (mean value of 0.79 for exocarp color, 0.70 for mesocarp color, and 0.63 for testa color). There was a low correlation between different traits in the same environment, with *R* value from 0.23 to 0.58 at a significant level (*P* < 0.01). The exocarp color showed higher correlation between mesocarp color than between testa color at Guiyang and Ledong in 2020, with mean *R* value of 0.55 and 0.28, respectively. However, the relationship between tannin content and exocarp and mesocarp color was higher than that of testa color.

## Genome-wide association study for pericarp color, testa color and tannin content

**Fig. 6:**
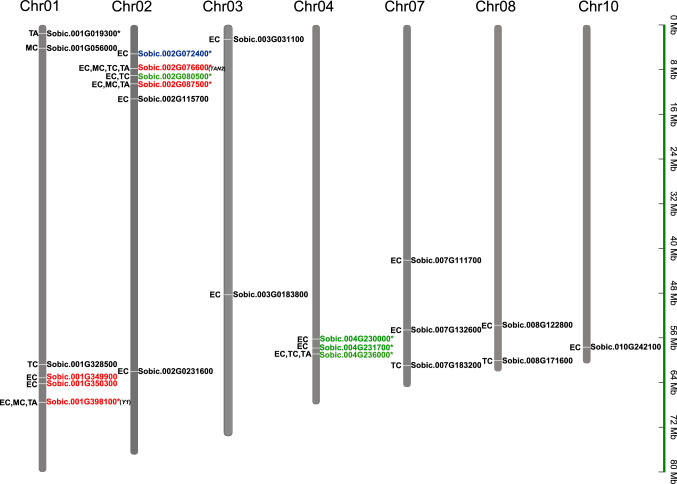
Schematic genetic map of important QTL identified for exocarp color (EC), mesocarp color (EC), testa color (TC), and tannin content (TA). Genes detected in 1, 2, 3 and 4 environments are shown in black, blue, green and red colors, respectively, while those identified by more than 2 models (including 2 models) are followed by asterisk (color figure online)

To investigate the genetic basis of natural variation in *sorghum* grain color and tannin contents, we conducted GWAS using 2,760,264 SNP markers with multiple models including Blink, FarmCPU, GLM, and MLM in GAPIT package. Totally, the four models identified 45, 12, 20 and 15 QTL for the color of exocarp, mesocarp, testa, and tannin content, respectively, involving in 73 QTL (Table 1, Fig. 6, Supplementary Table 2 and Figs. 2, 3). The QTL identified by Blink and FarmCPU were located on all chromosomes except for chromosome 5, while those identified by GLM and MLM were located on chromosomes 1, 2 and 4.

**Table 1:** Genes in or near important QTL obtained in present GWAS, corresponding orthologous genes, and their putative functions

QTL ID	Gene in or near QTL	Orthologous species	Orthologous Gene	Similarity (%)	Putative Function	References
qtl1.1602446	Sobic.001G019300*	*Oryza sativa*	LOC_Os03g62370	89.20	WD40-like Beta Propeller Repeat family protein	
qtl1.4286318	Sobic.001G056000	*Arabidopsis thaliana*	AT5G35550 (*TT2*)	62.70	MYB-like DNA-binding protein	Baudry et al. (2004)
qtl1.61599835	Sobic.001G328500*	*Arabidopsis thaliana*	AT5G41220	48.40	Glutathione S-transferase	Wagner et al. (2002)
qtl1.64009126	Sobic.001G349900*	*Arabidopsis thaliana*	AT5G07990 (*TT7*)	61.60	Favonoid 3'-monooxygenase	Kerhoas et al. (2006)
	Sobic.001G350300*	*Arabidopsis thaliana*	AT4G20970	53.70	bHLH DNA-binding superfamily protein	
qtl1.68376954	Sobic.001G398100*	*Sorghum bicolor*	*Y1*	100	MYB transcription factor	Ibraheem et al. (2010); Nida et al. (2019); Tao et al. (2021a, b)
qtl2.7379337	Sobic.002G072400*	*Arabidopsis thaliana / Oryza sativa*	AT3G04870 / *OsZDS*	90.70/94.80	Zeta-carotene desaturase	Dong et al. (2007); Fang et al. (2008)
qtl2.7983859	Sobic.002G076600*	*Sorghum bicolor*	*TAN2*	100	bHLH transcription factor	Wu et al. (2019); Xie et al. (2019)
qtl2.8464859	Sobic.002G080500*	*Arabidopsis thaliana*	AT5G23260 (*TT16*)	69.70	MADS box protein	Nesi et al. (2002)
qtl2.9341745	Sobic.002G087500	*Arabidopsis thaliana*	AT3G51240 (*TT6*) /AT4G22880 (*TT18*)	52.80/54.80	Flavanone 3-hydroxylase / leucoanthocyanidin dioxygenase	Shikazono et al. (2003); Wisman et al. (1998)
qtl2.13453215	Sobic.002G115700	*Arabidopsis thaliana*	AT5G13930 (*TT4*)	62.00	Chalcone synthase	Nakayama et al. (2019)
qtl2.62771164	Sobic.002G231600	*Zea mays*	Zm00001d032969 (*BZ2*)	56.60	Glutathione S-transferase	Zhao and Dixon (2010)
qtl3.2837597	Sobic.003G031100*	*Arabidopsis thaliana / Oryza sativa*	AT5G63650 (*EGL1*) / *OsbHLH025*	70.40/66.70	bHLH transcription factor	Nemie-Feyissa et al. (2015); Yamamura et al. (2015)
qtl3.48904117	Sobic.003G183800*	*Arabidopsis thaliana / Oryza sativa*	AT5G35550 (*TT2*) / *MYB61*	75.40	MYB transcription factor	Baudry et al. (2004); Gao et al. 2020
qtl4.57984828	Sobic.004G230000*	*Arabidopsis thaliana*	AT5G41220	51.00	Glutathione S-transferase	Wagner et al. (2002)
	Sobic.004G231700	*Arabidopsis thaliana*	AT5G35550 (*TT2*)	76.20	MYB transcription factor	Baudry et al. (2004)
qtl4.58261025	Sobic.004G236000	*Arabidopsis thaliana*	AT5G48100 (*TT10*)	70.20	Laccase/Diphenol oxidase family protein	Liang et al. (2006)
qtl7.41302381	Sobic.007G111700	*Arabidopsis thaliana*	AT5G17220 (*TT19*)	51.20	Glutathione S-transferase	Wisman et al. (1998)
qtl7.55281957	Sobic.007G132600*	*Arabidopsis thaliana*	AT5G35550 (*TT2*)	73.80	MYB transcription factor	Baudry et al. (2004)
qtl7.61701838	Sobic.007G183200*	*Arabidopsis thaliana*	AT4G09820 (*TT8*)	75.50	bHLH transcription factor	Petridis et al. (2016)
qtl8.54420851	Sobic.008G122800	*Arabidopsis thaliana*	AT5G07990 (*TT7*)	46.30	Flavonoid 3'-monooxygenase	Kerhoas et al. (2006)
qtl8.60826776	Sobic.008G171600	*Arabidopsis thaliana*	AT3G59030 (*TT12*)	80.00	MATE transporter	Marinova et al. (2007)
qtl10.58403535	Sobic.010G242100*	*Arabidopsis thaliana*	AT5G07990 (*TT7*)	46.50	Flavonoid 3'-monooxygenase	Kerhoas et al. (2006)

Gene within the QTL confidence interval is followed by an asterisk

### GWAS for exocarp color

**Fig. 7:**
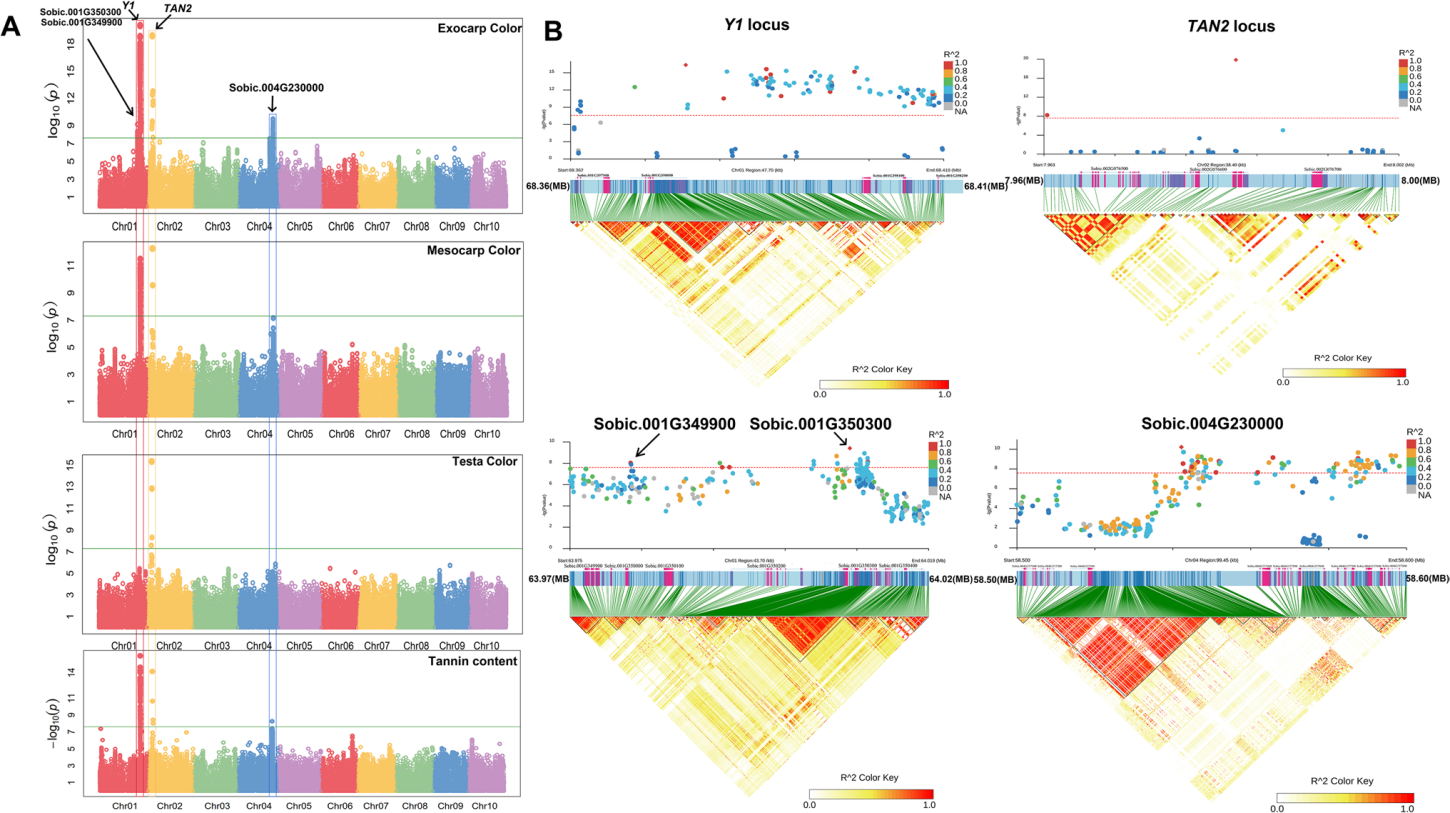
GWAS results for several important QTL on chromosomes 1, 2, and 4.** A** Manhattan plots for color of exocarp, mesocarp, and testa based on phenotype data in Guiyang in 2020, using GLM model. **B** Local Manhattan plot of *Y1*, *TAN2*, Sobic.001G349900, Sobic.001G350300, and Sobic.004G230000 and linkage disequilibrium (LD) heat map (color figure online)

Totally, 45 QTL were identified on all chromosomes except for chromosome 5. Two important QTL (qtl1.68376954 and qtl2.7983859) significantly associated with exocarp color were detected at the previously reported *Y1* locus on Chromosome 1 (Sobic.001G398100) and *TAN2* locus on Chromosome 2 (Sobic.002G076600), respectively. Another significant QTL (qtl1.64009126) was detected at the vicinity of *Y1* locus on chromosome 1 across all environments (Fig. 7A). The QTL (qtl1.64009126) was colocalized with two genes (Sobic.001G349900 and Sobic.001G350300), which fell into a high LD block (Fig. 7A, B, Table 1). The former was orthologous to *TT7* (AT5G07990) encoding flavonoid 3'-monooxygenase and the latter to the helix-loop-helix (bHLH) DNA-binding protein in *Arabidopsis* and rice. Moreover, three QTL were identified near the *TAN*2 locus on chromosome 2. One QTL (qtl2.7379337) colocalized with a gene (Sobic.002G072400) orthologous to both genes AT3G04870 in *Arabidopsis* and *OsZDS* in rice, which encodes zeta-carotene desaturase involved in the biosynthesis of carotenes and xanthophylls that reduces zeta-carotene to lycopene. Another QTL (qtl2.8464859) colocalized with a gene (Sobic.002G080500) orthologous to *TT16* (AT5G23260) encoding MADS box protein in *Arabidopsis* (Table 1, Fig. 6, Supplementary Fig. 4).

Two important QTL (qtl3.2837597 and qtl3.48904117) on chromosome 3 colocalized with two genes (Sobic.003G031100 and Sobic.003G183800) which were orthologous to AT5G63650 (*EGL1*)*/OsbHLH025* and AT5G35550 (*TT2*) */MYB61*, respectively, in *Arabidopsis* and rice, encoding bHLH transcription factor and MYB transcription factor (Table 1, Fig. 6, Supplementary Fig. 4).

In three environments in Guiyang (2018–2020), the three model (GLM, Blink, and FarmCPU) detected two significant QTL (qtl4.57984828 and qtl4. 58261025) on chromosome 4, which were close to reported *TAN1* locus (62.31 Mb). Three gene (Sobic.004G230000, Sobic.004G231700, and Sobic.004G236000) in or near the above two QTL encode orthologs of glutathione S-transferase (Fig. 7A, B), MYB transcription factor and laccase-like polyphenol oxidases, respectively. Moreover, three QTL (qtl7.55281957, qtl8.54420851, and qtl10.58403535) were detected on chromosome 7, 8 and 10, respectively. qtl7.55281957 fell into a LD block including a gene (Sobic.007G132600) orthologous to *TT2* (AT5G35550), while qtl8.54420851 and qtl10.58403535 colocalized with the genes Sobic.008G122800 and Sobic.010G242100, respectively, which both were orthologous to *TT7* (AT5G07990) (Table 1, Fig. 6).

### GWAS for mesocarp color

A total of 12 QTL were identified on chromosomes 1 (5), 2 (6), 8 (1) and 10 (1). Consistent with the results of the exocarp, GWAS based on the mesocarp color also detected *Y1* locus (qtl1.68376954) and *TAN2* locus (qtl2.7983859). Similarly, the QTL (qtl1.68757235) was again detected near *Y1* locus on chromosome 1. Meanwhile, two QTL were detected on chromosome 2 (qtl2.46205421) and chromosome 8 (qtl8.48591962), respectively (Table 1, Fig. 6).

### GWAS for testa color

Totally, 20 QTL were identified on the remaining seven chromosomes except for chromosomes 3, 5 and 10. Compared with the results of exocarp and mesocarp, GWAS based on the testa color only detected *TAN2* locus (qtl2.7983859) using the four models. Similarly, the QTL (qtl2.8300524) was again detected near *TAN2* locus, which is consistent with the results of the exocarp and mesocarp. Two QTL (qtl2.8464859 and qtl2.9115870) were identified at the vicinity of *TAN2* locus, as the same as the results of exocarp. Moreover, two related QTL (qtl7.61701838 and qtl7.62672940) were on chromosome 7, the former colocalized with a gene (Sobic.007G183200) orthologous to *TT8* (AT4G09820) (Table 1, Fig. 6).

### GWAS for tannin content

Totally, 15 QTLs were identified on the remaining seven chromosomes except for chromosomes 5, 6 and 8. Consistent with the results of the exocarp and mesocarp color, GWAS based on tannin content also detected *Y1* locus (qtl1.68376954) and *TAN2* locus (qtl2.7983859). The qtl2.8300524 was repeatedly detected near *TAN2* locus, consistent with the results for the color of epicarp, mesocarp and testa. A QTL (qtl1.1602446) on chromosome 1 was identified in Guiyang in 2020, corresponding a gene (Sobic.001G019300) whose orthologue encodes putative WD40-like protein in rice (Table 1, Fig. 6).

### Haplotype analysis of *Y1 *and *TAN2*

**Fig. 8:**
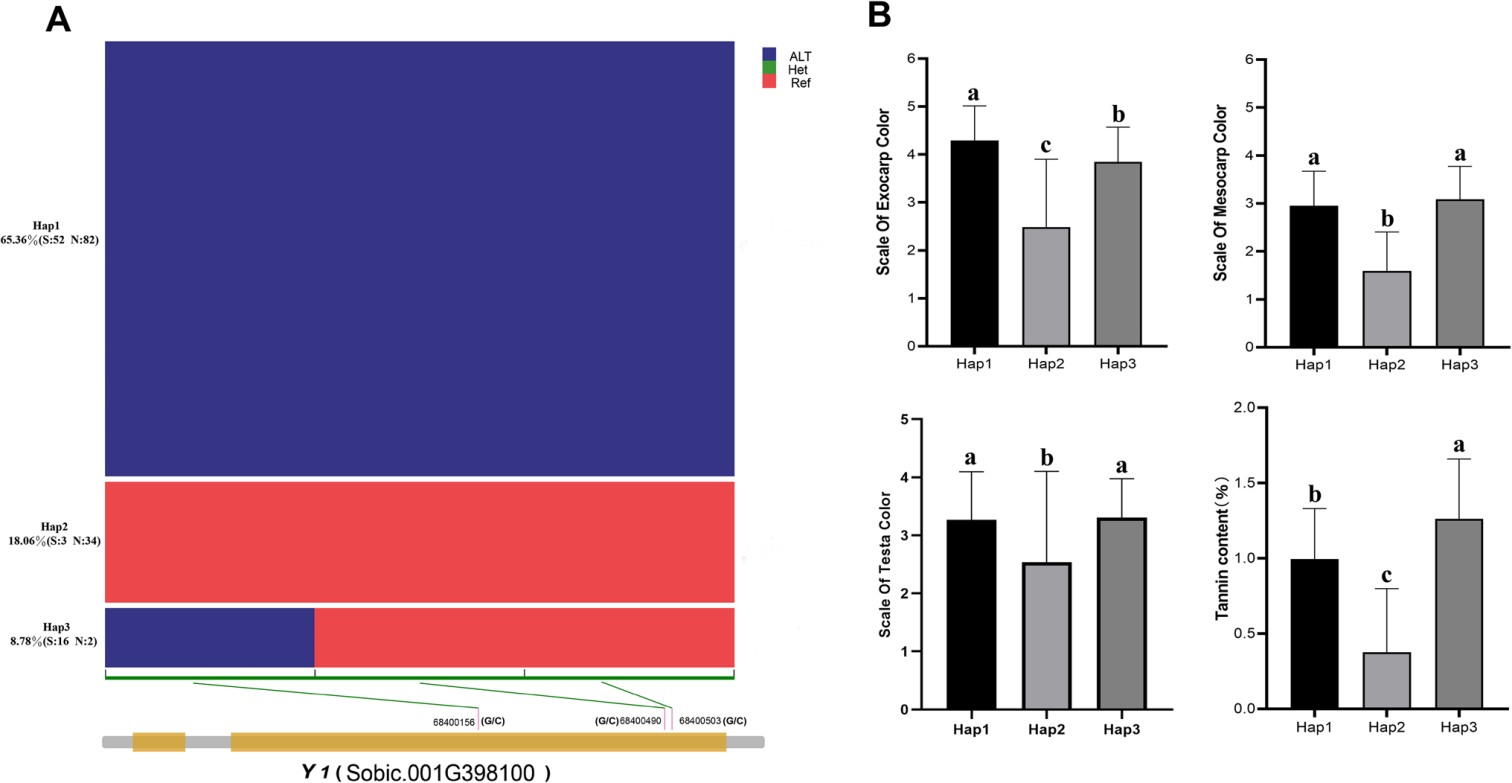
Haplotype analysis of *Y1 gene* and phenotype distribution for three haplotypes. **A** Three haplotypes of *Y1 gene* (Sobic.001G398100) and their frequencies in *sorghum* accessions. A schematic diagram of *Y1 gene* is presented at the bottom, and the positions (SNP) of mutant loci are marked. **B** Distributions of exocarp color, mesocarp color, testa color, and tannin content for the three haplotypes. Different letters denote significant differences according to the Tukey–Kramer test (*P* < 0.05). The error bars represent standard deviation (color figure online)

To investigate the genes mutation of *Y* and *TAN2* in Chinese *sorghum*, we performed haplotype analysis. For *Y1* gene, only three SNP mutant loci on the second exons were observed in Chinese population based on our genotyping data (Fig. 8A). All three SNP (G/C) variants were nonsynonymous mutations, located at the position of 68,400,156 bp, 68,400,490 bp and 68,400,503 bp, respectively, and resulted in amino acid change from alanine to proline, serine to threonine, and glutamine to histidine. The three main haplotypes were formed based on the three SNP mutations. More than 65% (134/242) of accession had three mutation SNPs and formed the first haplotype (Hap1), of which the northern and southern *sorghum* accounted for 61.94% and 38.81%, respectively. More than 18% (37/242) of *sorghum* had no mutant SNP, almost all come from northern China, forming the second haplotype (Hap2), while 8.78% (18/242) of accessions with the first mutant SNP formed the third haplotype (Hap3), 90% of which was from southern China. The accessions of Hap2 had light-colored grains and lowest tannin content, which was markedly different from the accessions of Hap1 and Hap3, while Hap3 accessions had the highest tannin content, which was inconsistent with the exocarp color distribution (Fig. 8B).

**Fig. 9:**
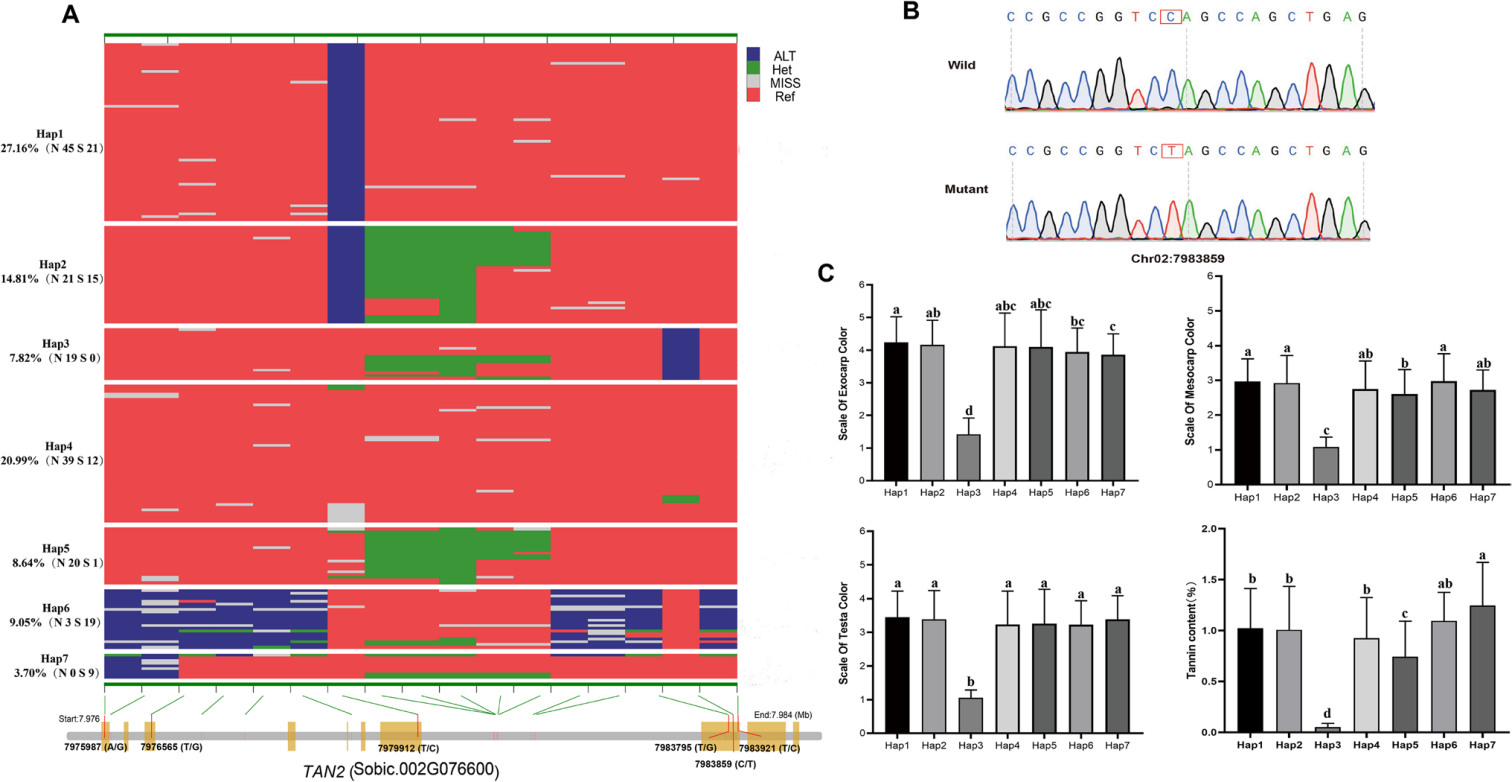
Haplotype analysis of *TAN2* gene and phenotype distribution for seven haplotypes. **A** Seven haplotypes of *TAN2* gene (Sobic.002G076600) and their frequencies in *sorghum* accessions. A schematic diagram of *TAN2* gene is presented at the bottom, and the positions (SNP) of mutant loci are marked. **B** Gene sequence comparison of the SNP mutant (C/T) in the 8th exon that cause premature transcription termination. **C** Distributions of exocarp color, mesocarp color, and tannin content for the seven haplotypes. Different letters denote significant differences according to the Tukey–Kramer test *P* < 0.05. The error bars represent standard deviation

Compared with *Y1 gene*, *sorghum* *TAN2* gene has 17 mutation sites (Fig. 9A), of which six mutants were located on the first, third, seventh, and eighth exons. Among six SNP variations, three SNP variations are nonsynonymous mutations. SNP (T/C) at the position of 7,979,912 bp on the seventh exon resulted in amino acid change from tryptophan to arginine, SNP (T/G) at the position of 7,983,795 bp on the eighth exon resulted in amino acid change from histidine to glutamine, and the second SNP (C/T) at the position of 7,983,859 bp in the eighth exon caused premature transcription termination (Fig. 9B). Chinese *sorghum* formed seven main haplotypes based on the six SNP variations. Hap1 and Hap2 included 66 and 36 accessions with a mutant in the seventh exon, respectively. Hap3 is composed of 19 Northern accessions with colorless testa and white pericarp due to the stop code mutation (C/T) on the eighth exon. The accessions of Hap4 and Hap5 are mainly from North China, while those of Hap6 and Hap7 are almost from South China. Except for Hap3, the colors of exocarp, mesocarp and testa of the other haplotypes did not differ significantly. The distribution of tannin content in seven haplotypes was basically consistent with those of pericarp and testa color. The germplasm with Hap3 had no tannins, while those with Hap4 contains the most tannins (Fig. 9C).

## Discussion

### Population structure of Chinese *sorghum*

After natural and artificial selection, the short-day *sorghum*, originated in Africa, has adapted to the agricultural ecological environment in China and is cultivated across agroclimatic zones from the short-day area in the south to the long-day area in the north. As expected, two distinct genetic sub-populations were identified in this study according to geographic origin, referring to the northern and the southern groups, respectively. Almost all accessions from Shaanxi province in northern China were clustered in the southern *sorghum* which showed that Shaanxi *sorghum* is closely related to southern *sorghum* and has continuous gene exchange with northern *sorghum*. In structure and PCA analysis, accessions for brewing Maotai-flavor liquor in Chishui River Basin in southwest China are differentiated from those of southern *sorghum*, which indicates that Chishui *sorghum* has some special properties suitable for making Maotai-flavor liquor. In addition, a new cluster was differentiated from the *sorghum* in Shanxi province in Northern China at *K* = 12 and indicating different genetics characteristic from other northern *sorghum*s. For example, *sorghum* is used for brewing vinegar, besides making liquor in Shanxi Province (Yan and Yan 2004).

### Phenotypic variation in grain color, testa pigment and tannin content

Among the major cereal crops, only *sorghum* grain has abundant condensed tannins, which plays an important role in the production and application of *sorghum*. Tannin in *sorghum* grains has been shown to reduce the damage of crops by birds, prevent pre-harvest germination, molding, protect *sorghum* plants against diseases caused by fungi, bacteria, and viruses. More importantly, hydrolysis of tannins during brewing process produces syringic acid and syringaldehyde essential to the flavors of the liquors (Dykes 2019; Wu et al. 2012, 2019; Xie et al. 2019).

*Sorghum* in south China often grows in hot, humid regions and is vulnerable to bird predation, pre-harvest germination, and diseases. The local farmers of south China prefer to cultivate tannin varieties. Therefore, grain tannin content is a key trait in Chinese *sorghum* breeding. In general, the tannin content of *sorghum* grains increases with the darkening of the pericarp (Lu 1999). However, pericarp color and its intensity are not a reliable indicator of tannins in *sorghum*s (Boren and Waniska 1992). It is a misconception that all *sorghum*s with a red or brown pericarp have tannins (Dykes and Rooney 2006). *Sorghum*s with a white, lemon-yellow, red, brown, or black pericarp may or may not have tannins depending upon the presence of a pigmented testa (Dykes et al. 2013). Likewise, our results revealed that tannin content had a low correlation with pericarp color, with R value ranging from 0.46 to 0.53, suggesting that breeding tannin varieties based on grain pericarp color is likely to lead to high deviations. We also observed a lower correlation between testa color and tannin content, which may be due to the relationship between tannin content and testa thickness. For tannin *sorghum*, varieties with lighter-pigmented, thicker testa might have higher tannin content than those darker-pigmented, thinner testa. In addition, almost all southern *sorghum* is tannin varieties, while all non-tannin varieties are planted in northern regions because *sorghum* is also used for food in the north, and tannins often contribute to the poor palatability of *sorghum* foods.

### GWAS results for known loci in *sorghum* tannin biosynthesis

Numerous genes involved in anthocyanin and tannin biosynthesis are well studied in maize, rice, and *Arabidopsis* (Chatham et al. 2019; Petroni and Tonelli 2011). The genes involved in anthocyanin biosynthesis can be classified into two categories of structural and regulatory genes. Structural genes encode functional enzymes that catalyze anthocyanin-biosynthesis reactions, and regulatory genes encode mainly transcription factors that regulate the expression of structural genes (Petroni and Tonelli 2011). To date, only three regulatory genes involved in tannin biosynthesis have been identified in *sorghum*, no structural genes for tannin biosynthesis have been identified. Obviously, there is a huge gap between *sorghum* and other crops in the genetic mechanism of tannin synthesis pathway.

To accurately map QTL that are significantly associated with grain color and tannin content, we used four models (Blink, FarmCPU, GLM, and MLM) to perform GWAS with > 2 million SNPs obtained from the whole-genome sequencing data. In general, all four models detected the known loci on chromosomes 1, and 2, while Blink and FarmCPU were able to find additional new important QTL other than the loci mentioned above. GLM and MLM usually detect those loci with major effect, while Blink and FarmCPU are able to identify loci with minor effect. This indicated that certain loci that have a significant contribution to the trait of interest may be ignored, remain undiscovered or become marginal in some GWAS models. Therefore, it is critical to use multiple models to improve the detection power and robustness of GWAS (Chang et al. 2018; Li et al. 2014; Nida et al. 2021a; Xu et al. 2018).

In this study, the known genes (*Y1* and *TAN2*) that control the biosynthesis of grain color and tannin were detected in all environments using four models. The pigmented testa is controlled by *TAN1* and *TAN2* genes (Wu et al. 2012, 2019). It requires the presence of both dominant *TAN1* and *TAN2* to synthesize condensed tannins and to have a pigmented testa. Strangely, the *TAN1* gene on chromosome 4 was not detected in our collection. Contrary to the results reported in GWAS of the *sorghum* diverse panels from Africa and America. The gene *TAN1* rather than *TAN2* was detected in Ethiopian *sorghum* germplasm (Nida et al. 2021a), *sorghum* association panel (SAP) (Rhodes et al. 2014, 2017), as well as global *sorghum* collection (Tao et al. 2021a). The most likely explanation for these discrepancies is that the minor allele frequency (MAF) of *TAN1* or *TAN2* was less than 0.05 in the *sorghum* diversity panels resulting in no significant signal detected in GWAS.

The *Y1* gene is one of two important genes that controls the pericarp color of *sorghum* kernel (Dykes 2019; Dykes and Rooney 2006). In our collection, the *Y1* gene formed three haplotypes, indicating that the gene sequence is relatively conserved in Chinese *sorghum*. Varieties with Hap1 and Hap3 showed red or brown pericarps, while varieties with Hap2 showed white or yellow pericarps. The previously studies reported a 3.2-kb deletion in 4 *sorghum* lines, including the reference genome BTx623, which resulted in loss of *Y1* gene function (Nida et al. 2019; Tao et al. 2021a). However, we could not determine whether this deletion allele exists in our accessions by alignment analysis of the resequencing data with the reference genome (BTx623), or will carry out gene sequencing in our further studies if necessary. Therefore, it is important to select a more comprehensive reference genome when conducting grain color and tannin studies in the future, such as pan-genome.

Moreover, we identified seven haplotypes of *TAN2* controlling the pigmented testa (Wu et al. 2012, 2019). The accessions with Hap3 showed colorless testa and white pericarp because the SNP mutation (C/T) in exon 8 caused an early stop codon and formed a new recessive allelic variant of *TAN2* in Chinese *sorghum*. The previous study (Wu et al. 2019) also reported three recessive alleles *tan2-a*, *tan2-b* and *tan2-c* of *TAN2* in 180 global germplasms, corresponding to the 5-bp insertion in exon 7 (tan2-a), a 7-bp insertion in exon 7 (tan2-b) and a large deletion removing the entire 95-bp intron between exon 7 and exon 8 (tan2-c), respectively. Our findings suggested that breeders paid more attention to genetic variation at the *TAN2* locus to obtain new varieties with different tannin contents in Chinese *sorghum* breeding programs. Consequently, we should accelerate the improvement of Chinese *sorghum* varieties by introducing foreign germplasm resources containing superior allelic variation of *TAN1* locus, using molecular breeding techniques.

### Candidate regulator genes for tannin biosynthesis in *sorghum*

The anthocyanin regulatory system is conserved among species, requiring a ternary MYB-bHLH-WDR (MBW) complex (Chatham et al. 2019). In the *sorghum* proanthocyanidins pathway, the WD40 (*TAN1*) and bHLH (*TAN2*) are two components of the ternary MBW. So far, the third member of this complex, MYB transcription factor, has not been identified in *sorghum*. The Arabidopsis *TT2*, *TT8* and *TTG1* genes, encoding R2R3-MYB, bHLH and WD40 repeat domain proteins, respectively, are involved in the MYB-bHLH-WD40 (MBW) complex that activates proanthocyanidins in *Arabidopsis* seeds (Baudry et al. 2004). The *TT2* orthologue on chromosome 4 (Sobic.004G231700, 58.10 Mb, 76.2% similarity), which was located in a significant QTL (qtl4. 58545077) detected in three environments using three model, is most likely the candidate for the missing MYB. Moreover, other three *TT2* orthologs were identified on chromosome 1 (Sobic.001G056000, 62.7% similarity), 3 (Sobic.003G183800, 75.4% similarity), and 7 (Sobic.007G132600, 73.8% similarity), which are also possible candidate genes of the missing MYB. For example, the QTL (qtl3.48904117) on chromosome 3 corresponded to a gene (Sobic.003G183800) that was orthologous of *TT2* in *Arabidopsis* and *MYB61* in rice (Gao et al. 2020), encoding MYB family transcription factor. QTL containing or near to the above genes overlapped with the reported QTL for grain color and tannin content in previous *sorghum* studies (Guindo et al. 2019; Rhodes et al. 2014).

The intensifier gene *I* affects the intensity of the pericarp color and is most apparent in red *sorghum*s (Dykes 2019; Dykes and Rooney 2006). A recessive intensifier of anthocyanin biosynthesis in maize, *in1* (intensifier1), encodes a bHLH type protein with high sequence similarity to those in a ternary MYB-bHLH-WDR (MBW) complex, regulating pigmentation in vegetative tissue through competitive inhibition (Chatham et al. 2019). Our GWAS detected three significant QTL that are orthologous to genes encoding bHLH transcription factors in *Arabidopsis* and rice. The gene (Sobic.001G350300) in the important QTL (qtl1.64009126) detected in all environments, encoded the putative bHLH DNA-binding superfamily protein. The previously reported QTL for grain color were overlapped with this QTL (Zhang et al. 2015). In addition, a QTL (qtl3.2837597) on chromosome 3 colocalized with a gene (Sobic.003G031100) encodes a bHLH orthologous of *Arabidopsis EGL1* (AT1G63650) (Nemie-Feyissa et al. 2015) and rice *OsbHLH025* (Yamamura et al. 2015). Other QTL (qtl7.61701838) on chromosome 7 colocalized with gene (Sobic.007G183200) is orthologous of *Arabidopsis TT8* (AT4G09820) (Petridis et al. 2016) and was consistent with previously reported QTL for grain color in *sorghum* (Fernandez et al. 2008). Therefore, we speculate that these three loci are likely candidates for the* I* gene.

### Candidate structural genes for tannin biosynthesis in *sorghum*

It is known that structural genes involved in *sorghum* tannin synthesis remain unknown so far. In this study, we identified about 73 QTL associated with the color of exocarp, mesocarp, testa, and tannin content, besides *Y* and *TAN2* loci. Some of these loci are orthologous to structural genes in known tannin synthesis pathways in *Arabidopsis*, Maize, and rice.

The main enzymes that participate in the biosynthesis of anthocyanins include chalcone synthesis (CHS), flavanone 3-hydroxylase (F3H), flavonoid 3’-hydroxylase (F3’H), and leucoanthocyanidin reductase (LAR) (Li et al. 2022). The QTL (qtl2.13453215) on chromosome 2 was colocalized with Sobic.002G115700 that was orthologous to *TT4* (62.0% similarity) encoding chalcone synthase (CHS), a key enzyme involved in the biosynthesis of flavonoids in *Arabidopsis* (Nakayama et al. 2019). In addition, our GWAS identified three QTL that overlapped with the reported QTL for grain color in previous *sorghum* studies (Fernandez et al. 2008; Zhang et al. 2015). They colocalized with Sobic.001G349900 (63.98 Mb) on chromosome 1, Sobic.008G122800 (54.42 Mb) on chromosome 8 and Sobic.010G242100 (58.40 Mb) on chromosome 10. These genes are *Arabidopsis TT7* and maize *Pr1* orthologs encoding flavonoid 3'-monooxygenase, which is required for F3’H activity (Kerhoas et al. 2006; Li et al. 2022).

Two significant QTL for several traits identified in multi-environments were located in the vicinity of *TAN2* locus on chromosome 2. One important QTL (qtl2.9341745) corresponded to a gene (Sobic.002G087500), which was orthologous to *TT6* (52.8% similarity) and *TT18* (54.8% similarity) involving in the biosynthesis of two important enzymes F3H and LAR in *Arabidopsis* (Shikazono et al. 2003; Wisman et al. 1998). Another important QTL (qtl2.7379337) related to exocarp color was colocalized with gene (Sobic.002G072400), which was orthologous to *AT3G04870* (90.7% similarity) in Arabidopsis and *OsZDS* (94.8% similarity) in rice, involving in the biosynthesis of carotenes and xanthophylls, which reduces zeta-carotene to lycopene in *Arabidopsis* (Dong et al. 2007) and rice (Fang et al. 2008). These loci were also consistent with previously mapping QTL for tannin content and polyphenol content in *sorghum* (Marla et al. 2019; Rhodes et al. 2017).

In addition, a significant QTL (qtl2.8464859) on chromosome 2 associated with exocarp color and testa pigment in several environments colocalized with a putative *TT16* ortholog (Sobic.002G080500) that encodes a MADS box protein involving in regulate proanthocyanidin biosynthesis in the inner-most cell layer of the seed coat in *Arabidopsis* (Nesi et al. 2002). The important QTL (qtl4.58261025) on chromosome 4 significantly related to exocarp color, testa pigment and tannin content in all environments, included *TT10* orthology (70.20% similarity) encoding laccase-like polyphenol oxidases. It involved in lignin and flavonoids biosynthesis and expressed in developing testa, where it colocalized with the flavonoid end products proanthocyanidins and flavonols (Liang et al. 2006). The above two QTL overlapped with previously reported QTL related to proanthocyanidins (Rhodes et al. 2014), polyphenol content (Habyarimana et al. 2019), and grain color (Guindo et al. 2019; Zhang et al. 2015).

### Candidate transporter genes for tannin biosynthesis in *sorghum*

Because *S* gene controls the presence of brown pigments in the exocarp and endocarp when a pigmented testa is present (Blakely et al. 1979), tannin *sorghum* can be divided into two genotypes based on their genetics and chemical analyses (Dykes and Rooney 2006). *Sorghum*s with genotype of *TAN1*_*TAN2*_*ss* can synthesize tannins that are deposited in the vesicles within the testa layer, thus requiring acid (1% HCl methanol) to disrupt the structure of the vesicles to release tannins (Earp et al. 2004a). Whereas tannins *sorghum*s with genotype of *TAN1*_*TAN2*_*S*_ can be extracted with either methanol or acidified methanol because their tannins are deposited along the cell walls of the testa and some are present in the pericarp, consistent results were observed in our GWAS, and *TAN2* locus was significantly associated with exocarp, mesocarp and testa, while *Y1* locus was only significantly associated with epicarp and mesocarp. This indicated that tannins in the genotype (*TAN1_TAN2_S_*) were able to affect pericarp pigmentation, whereas tannins in the genotype (*TAN1_TAN2_ss*) did not affect the pericarp color, thus showing light pericarp and pigmented testa. Anthocyanins or proanthocyanidins are synthesized on the cytoplasmic interface of the endoplasmic reticulum and need to be transported to the vacuole for storage with the assistance of a glutathione S-transferase (GST) or MATE transporter in maize and *Arabidopsis* (Grotewold and Davies 2008; Ralston and Yu 2006).

In present study, three QTL were colocalized with the GST orthologous genes on chromosomes 1, 2 and 4. The significant QTL (qtl2.62771164) on chromosome 2 was close to gene (Sobic.002G231600, 62.30 Mb) encoding the *BZ2* ortholog in maize (Zhao and Dixon 2010). The other two genes (Sobic.001G328500 and Sobic.004G230000) encoded putative GST orthologs of AT5G41220 in *Arabidopsis* (Wagner et al. 2002). In addition, the QTL (qtl8.60826776) on chromosome 8 was at the vicinity of the gene (Sobic.008G171600, 60.54 Mb), which encodes an orthologous of the *Arabidopsis* MATE transporter *TT12*, controlling proanthocyanidins transport to the vacuole (Marinova et al. 2007). Two previous *sorghum* GWAS identified QTL related to tannin content and grain color that were close to the gene (Sobic.004G230000) in our research (Guindo et al. 2019; Rhodes et al. 2014). These reports lend additional support that the above loci are possible candidates for *S* gene.

In present study, we identified several potential candidate genes for tannin biosynthesis in *sorghum*. Accordingly, a targeted biparental mapping population may be more efficient to precisely identify the causal alleles. Subsequently, sequence analysis and expression analysis of these candidate genes are required to identify the causal polymorphisms and lay the foundation for the application of tannin genetic variation in crop improvement.

In summary, population structure analysis revealed that 242 Chinese *sorghum* accessions clustered into northern and southern subgroups based on their origin. Phenotypic survey showed that the correlation between tannin content and pericarp color was low, and tannin *sorghum* had better adaptability to the agroecology of southern China. GWAS identified 73 QTL for pericarp color, mesocarp color, testa pigment and tannin content, of which 47 might be novel QTL. Many important QTL were colocalized with orthologous of flavonoid pathway genes from other plants, of which several possible candidate genes were proposed as regulator, structural, and transporter genes for tannin biosynthesis in *sorghum*. Our results provided guidance for breeding *sorghum* suitable brewing Chinese liquors.

## Supplementary Information

Below is the link to the electronic supplementary material.

Supplementary file1 (XLSX 38 KB)

Supplementary file2 (XLSX 23 KB)

Linkage disequilibrium (LD) patterns of all (green), northern (red) and southern sorghums (blue) in China, with LD decay distance (*r*_2_ = 0.2) of 62.5 kb, 76.5 kb, and 59.7 kb, respectively, based on 2,760,264 SNP for 242 Chinese sorghum in this study (PDF 52 KB)

Manhattan plots for color of exocarp (EC), mesocarp (MC), and testa (TC), as well as tannin content (TA) based on phenotype data in Guiyang in 2018 (18GY), 2019 (19GY), 2020 (20GY), and Ledong in 2020 (20LD) using GLM and MLM model. The title of each small plot is represented by model_ environment_trait (PDF 739 KB)

Manhattan plots for color of exocarp (EC), mesocarp (MC), and testa (TC), as well as tannin content (TA) based on phenotype data in Guiyang in 2018 (18GY), 2019 (19GY), 2020 (20GY), and Ledong in 2020 (20LD) using Blink and FarmCPU model. The title of each small plot is represented by model_ environment_trait (PDF 736 KB)

Local Manhattan plot and LD heat map for important loci related to the color of exocarp, mesocarp and testa, as well as tannin content (PDF 47184 KB)

## Data Availability

The datasets presented in this study can be found in online repositories. The name of the repository and accession number can be found below: China National GeneBank (CNGB); CNP0002968. The website links are as follows: https://db.cngb.org/search/project/CNP0002968/.
