# Supplementary figures and images for: GWAS of grain color and tannin content in Chinese sorghum based on whole-genome sequencing
Source: Theor Appl Genet. 2023 Mar 23;136(4):77. doi: 10.1007/s00122-023-04307-z (PMC10036430; doi:10.1007/s00122-023-04307-z)

## LD decay

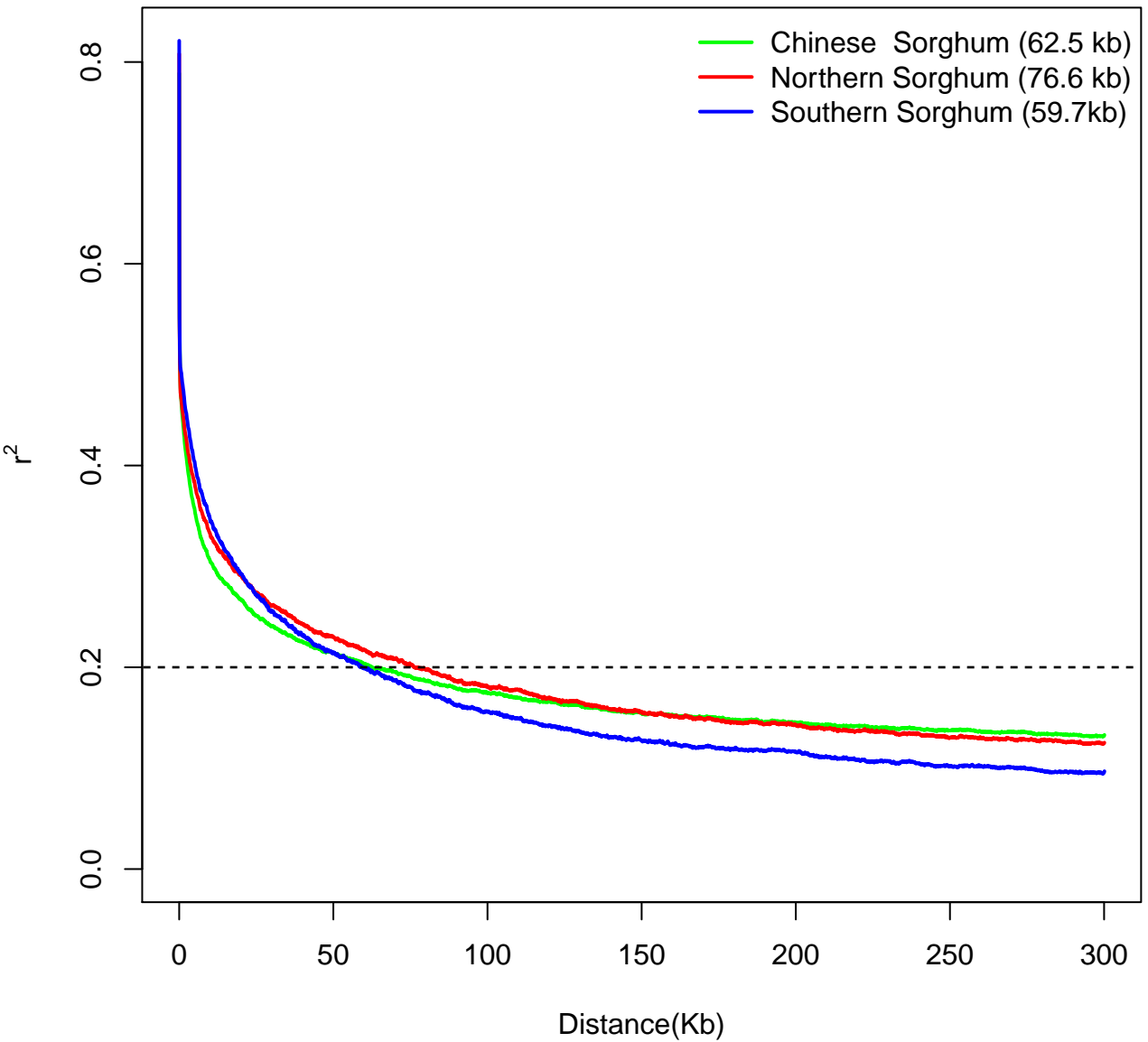

Supplement: Supplementary file 3 — Linkage disequilibrium (LD) patterns of all (green), northern (red) and southern sorghums (blue) in China, with LD decay distance (r2 = 0.2) of 62.5 kb, 76.5 kb, and 59.7 kb, respectively, based on 2,760,264 SNP for 242 Chinese sorghum in this study (PDF 52 KB) [file 122_2023_4307_MOESM3_ESM.pdf]

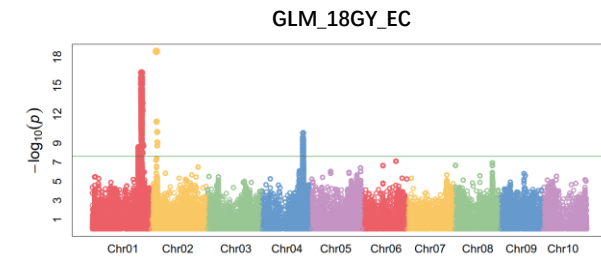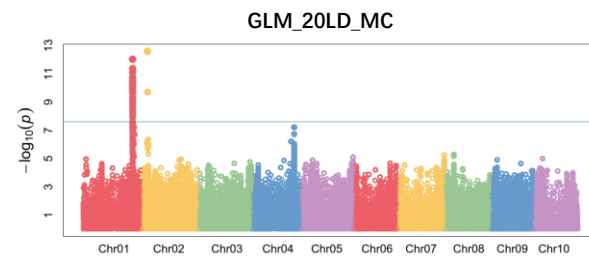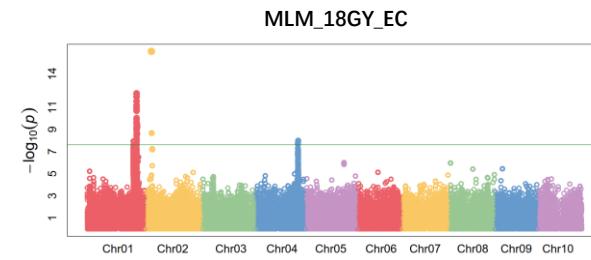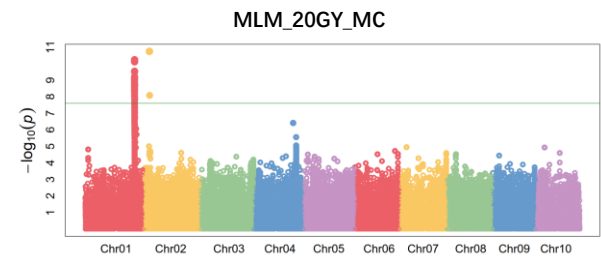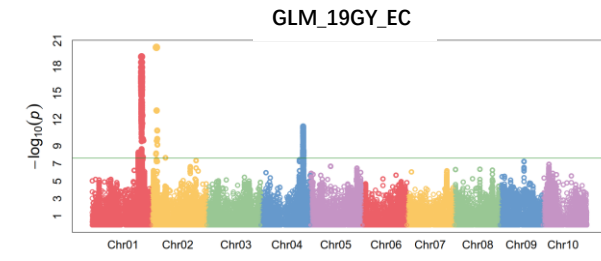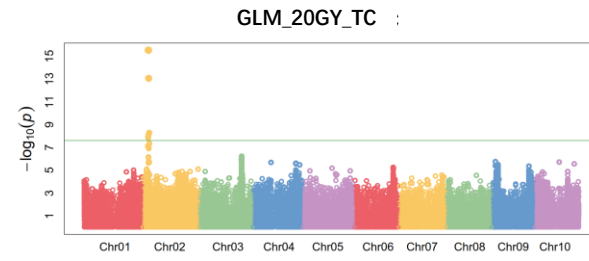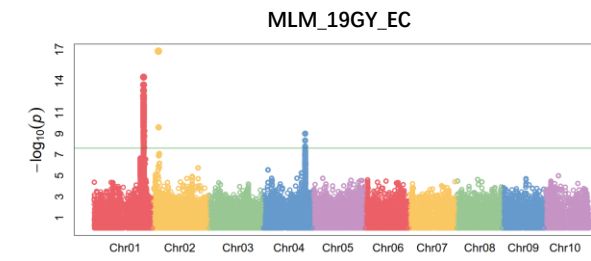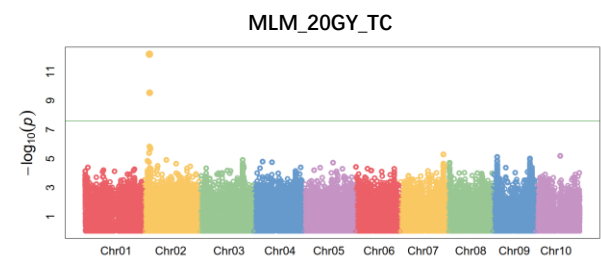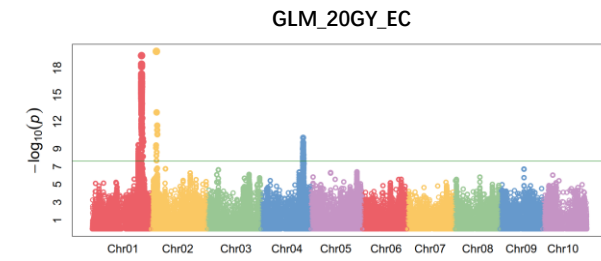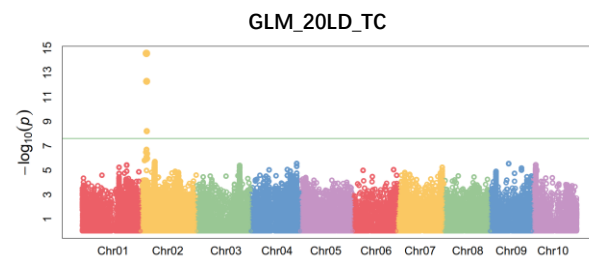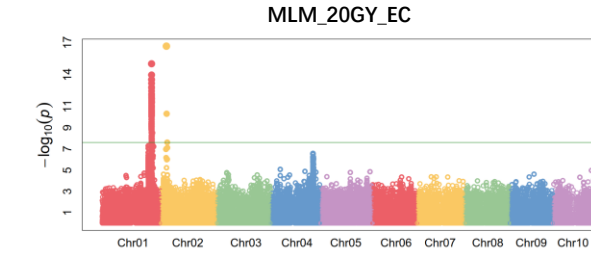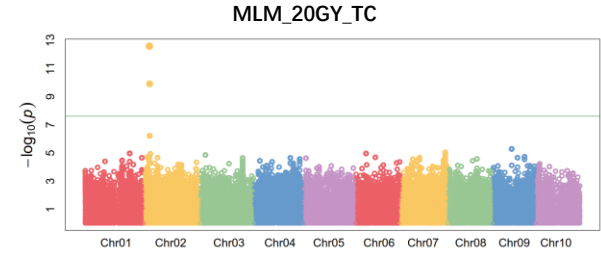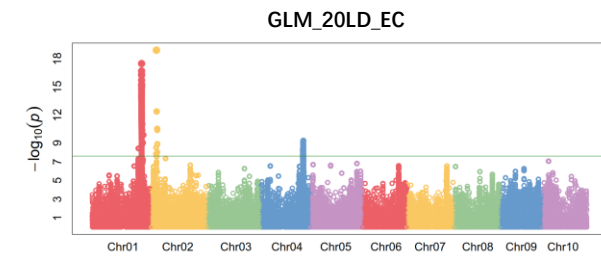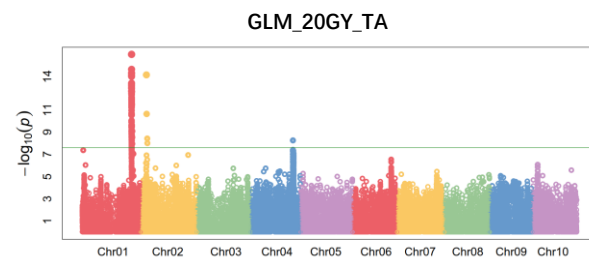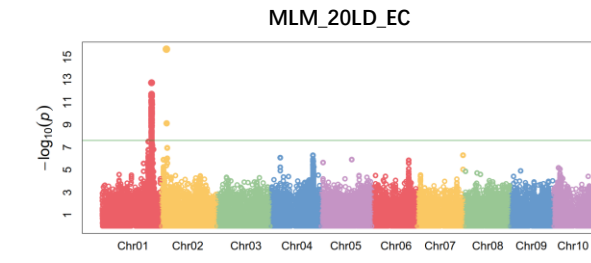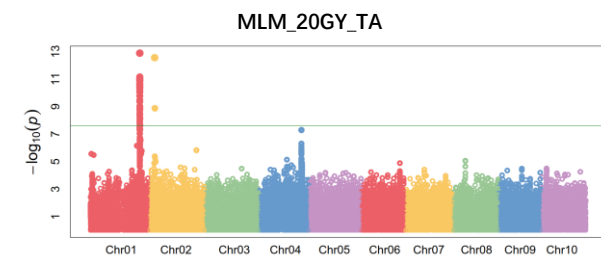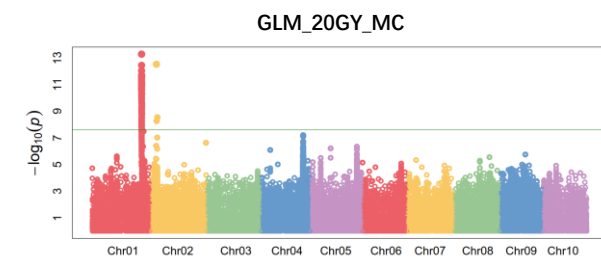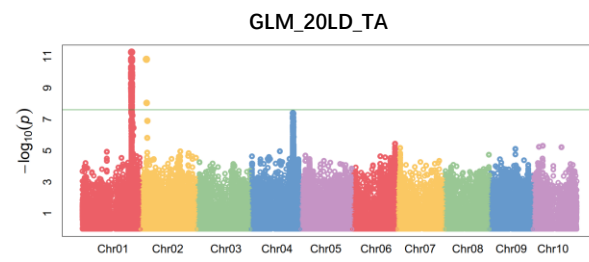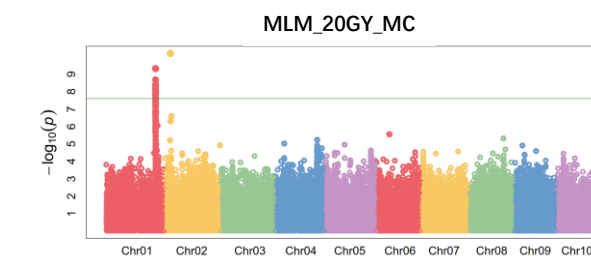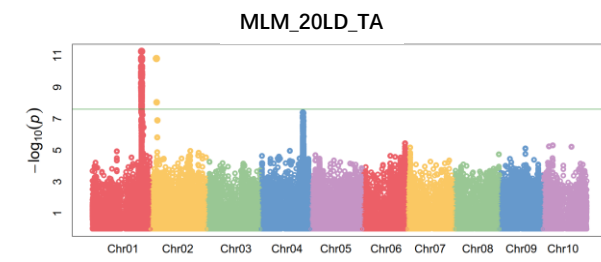

Supplement: Supplementary file 4 — Manhattan plots for color of exocarp (EC), mesocarp (MC), and testa (TC), as well as tannin content (TA) based on phenotype data in Guiyang in 2018 (18GY), 2019 (19GY), 2020 (20GY), and Ledong in 2020 (20LD) using GLM and MLM model. The title of each small plot is represented by model_ environment_trait (PDF 739 KB) [file 122_2023_4307_MOESM4_ESM.pdf]

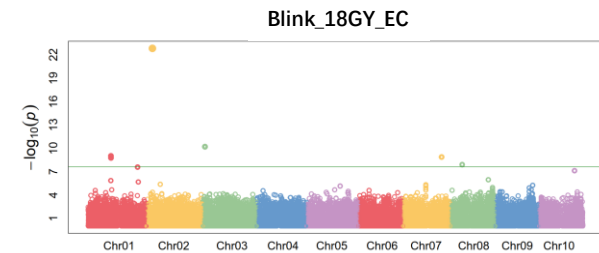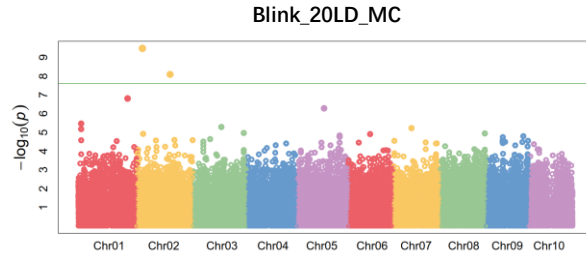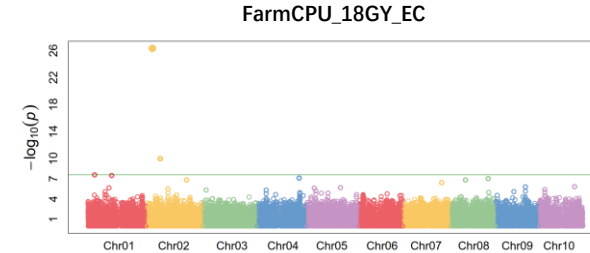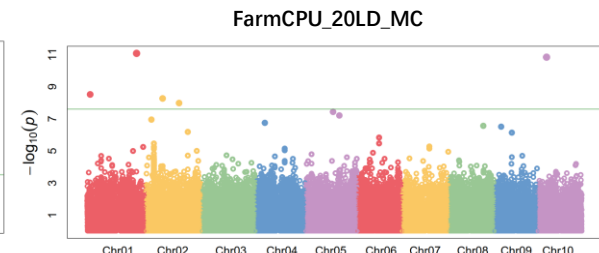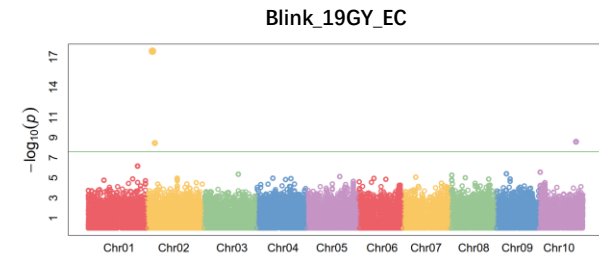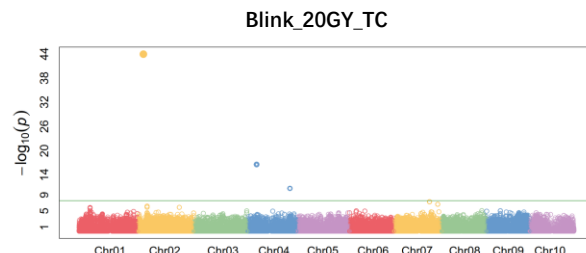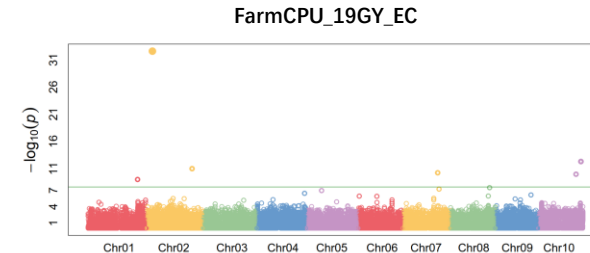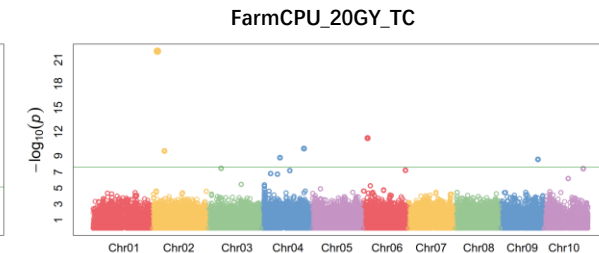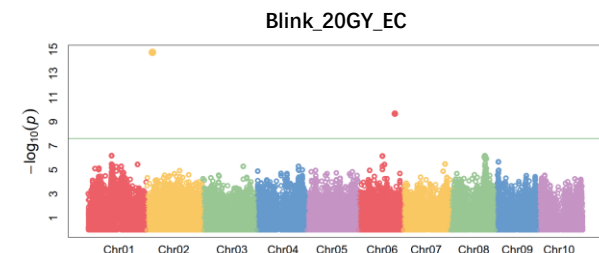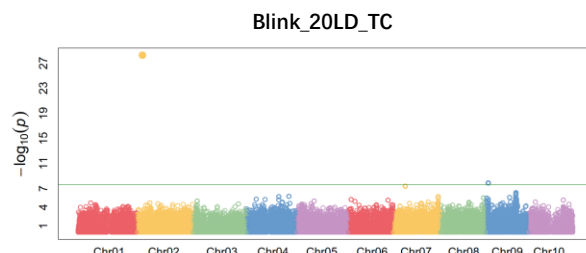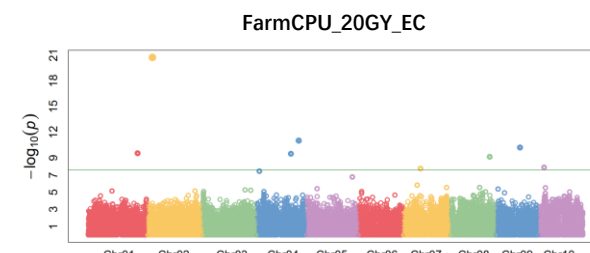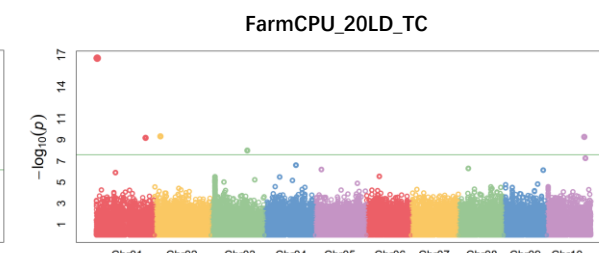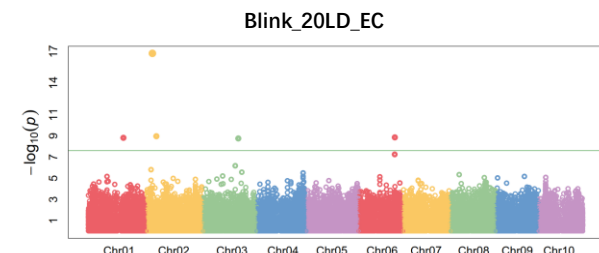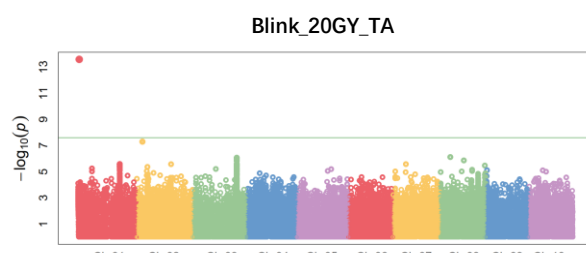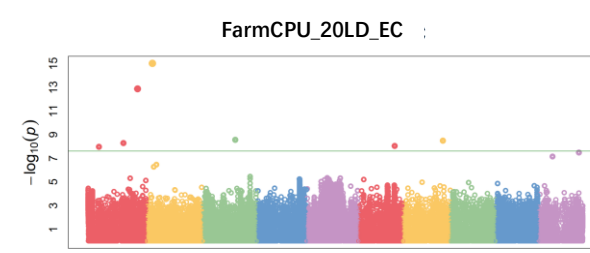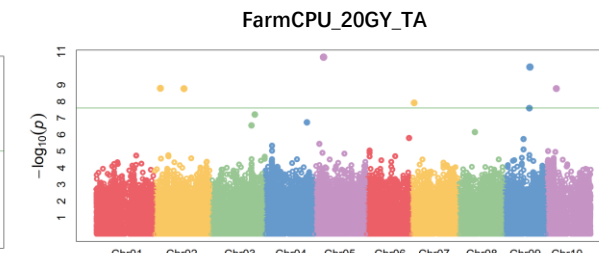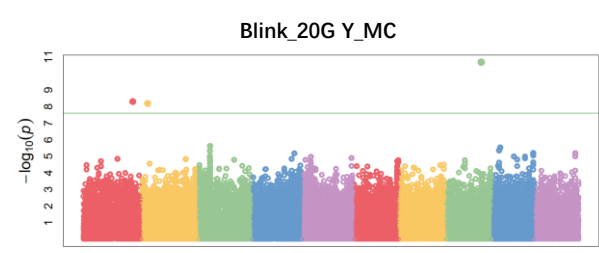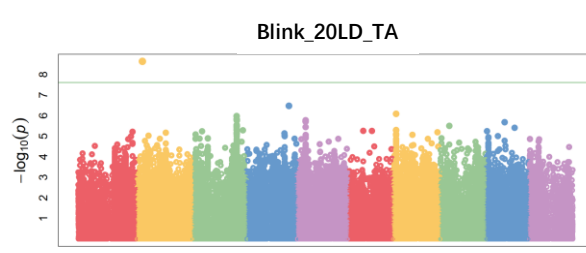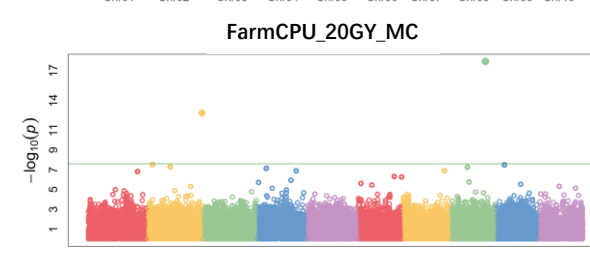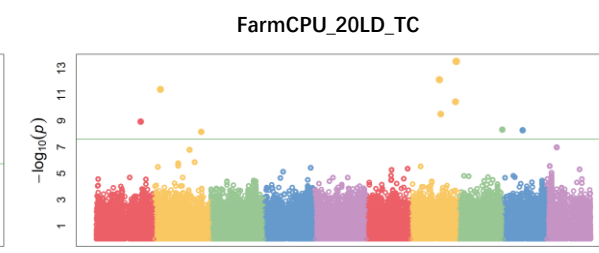

Supplement: Supplementary file 5 — Manhattan plots for color of exocarp (EC), mesocarp (MC), and testa (TC), as well as tannin content (TA) based on phenotype data in Guiyang in 2018 (18GY), 2019 (19GY), 2020 (20GY), and Ledong in 2020 (20LD) using Blink and FarmCPU model. The title of each small plot is represented by model_ environment_trait (PDF 736 KB) [file 122_2023_4307_MOESM5_ESM.pdf]

A

Sobic.002G072400

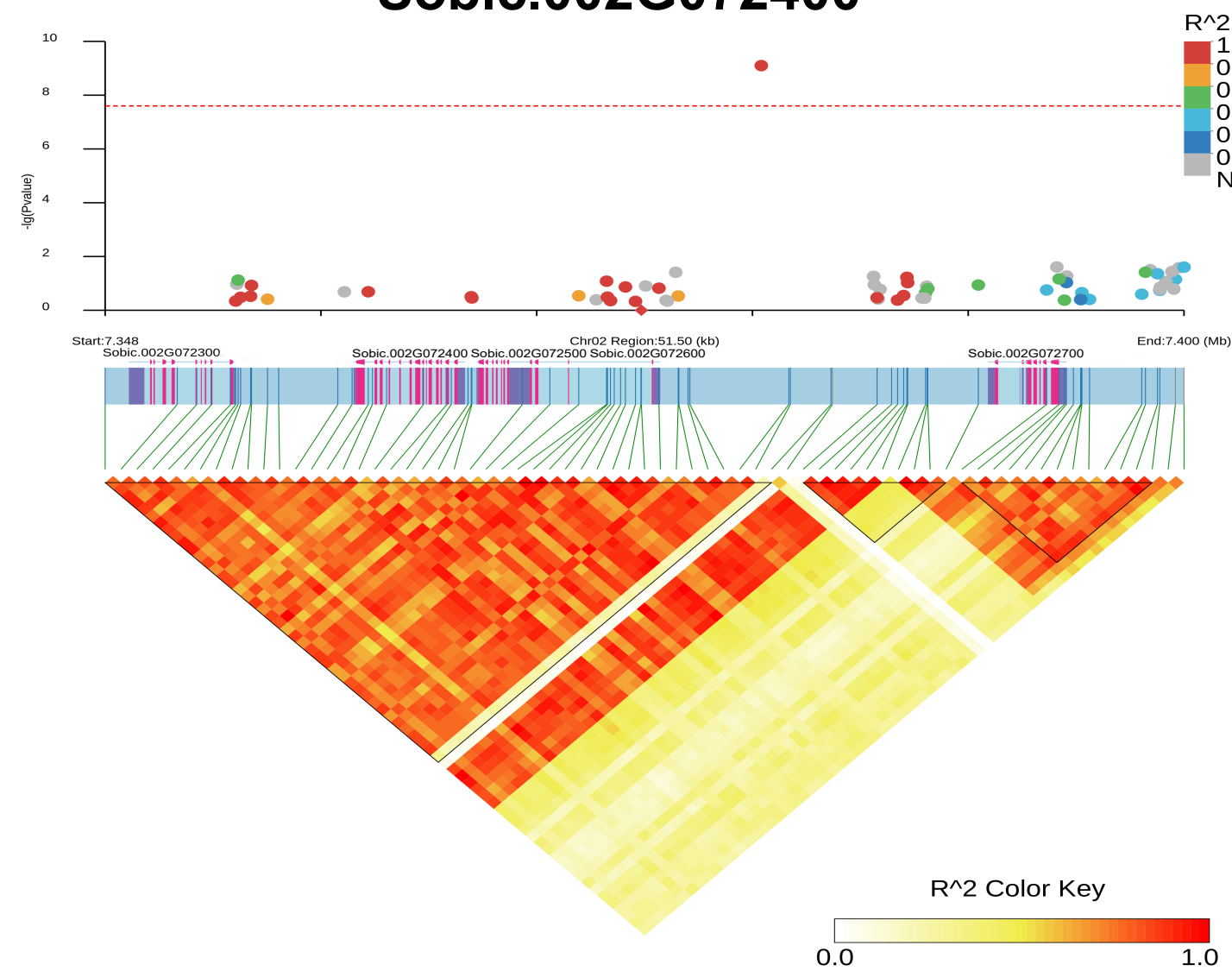

B

Sobic.002G080500

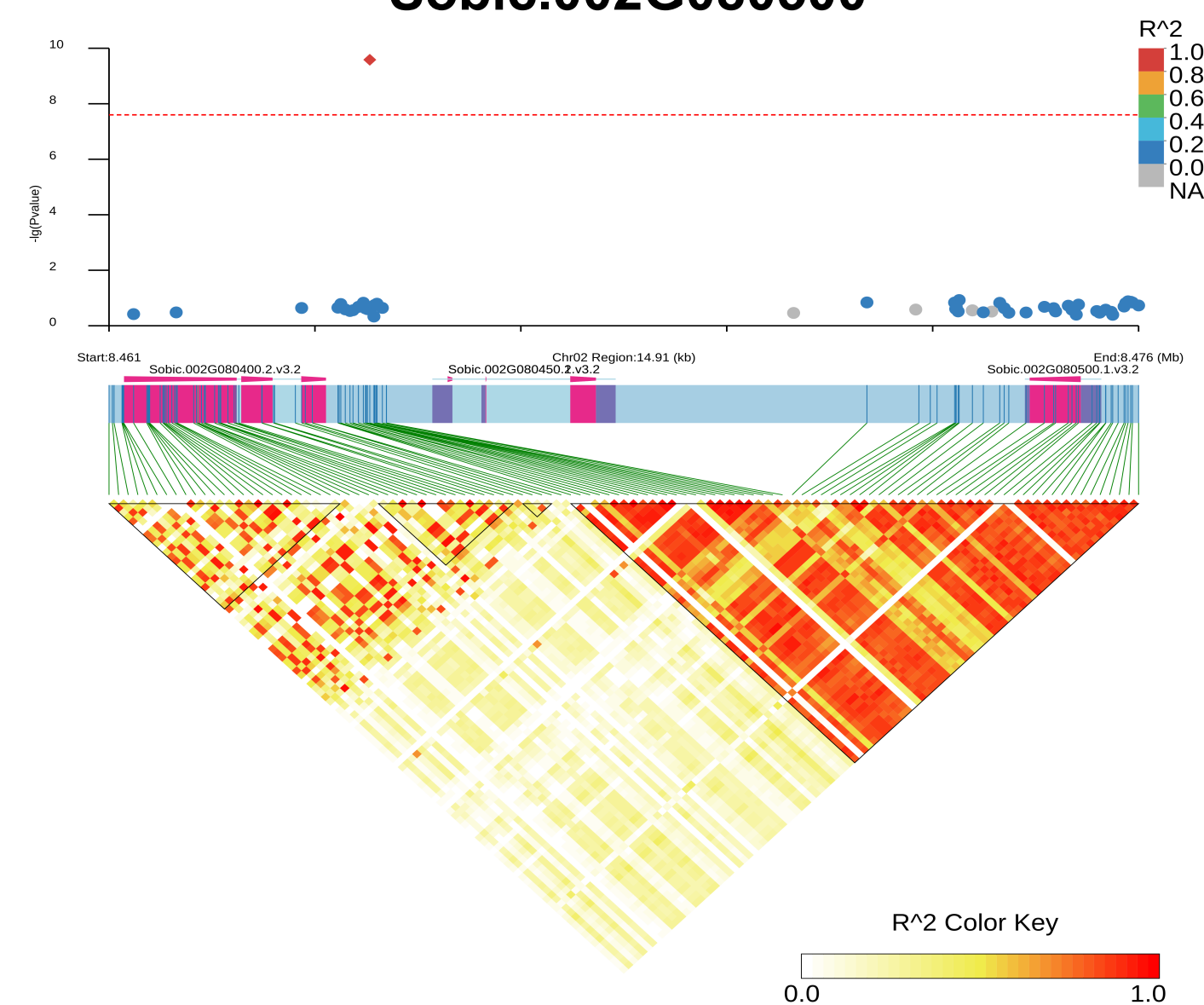

C

Sobic.003G031100

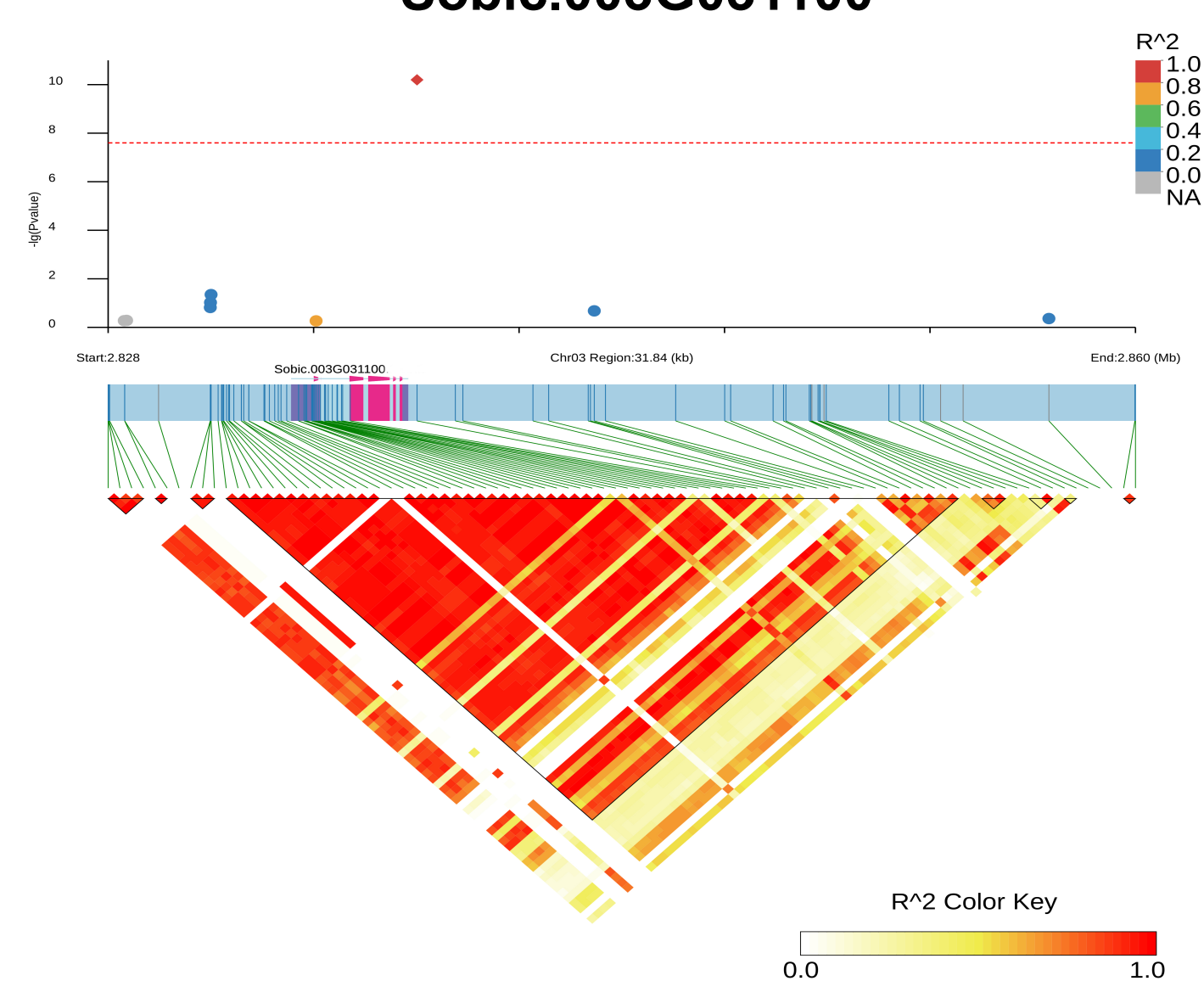

D

Sobic.003G183800

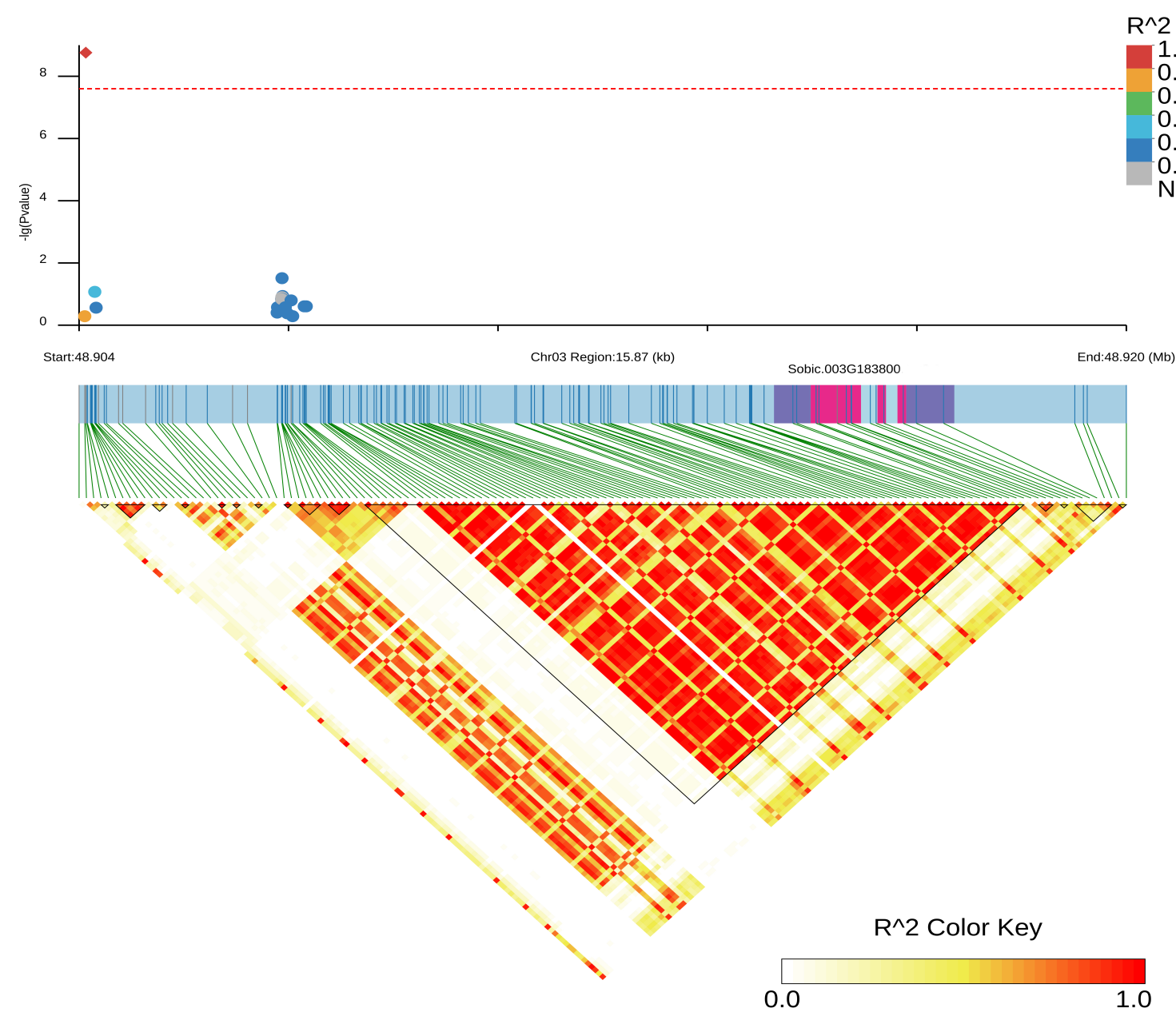

E

Sobic.007G132600

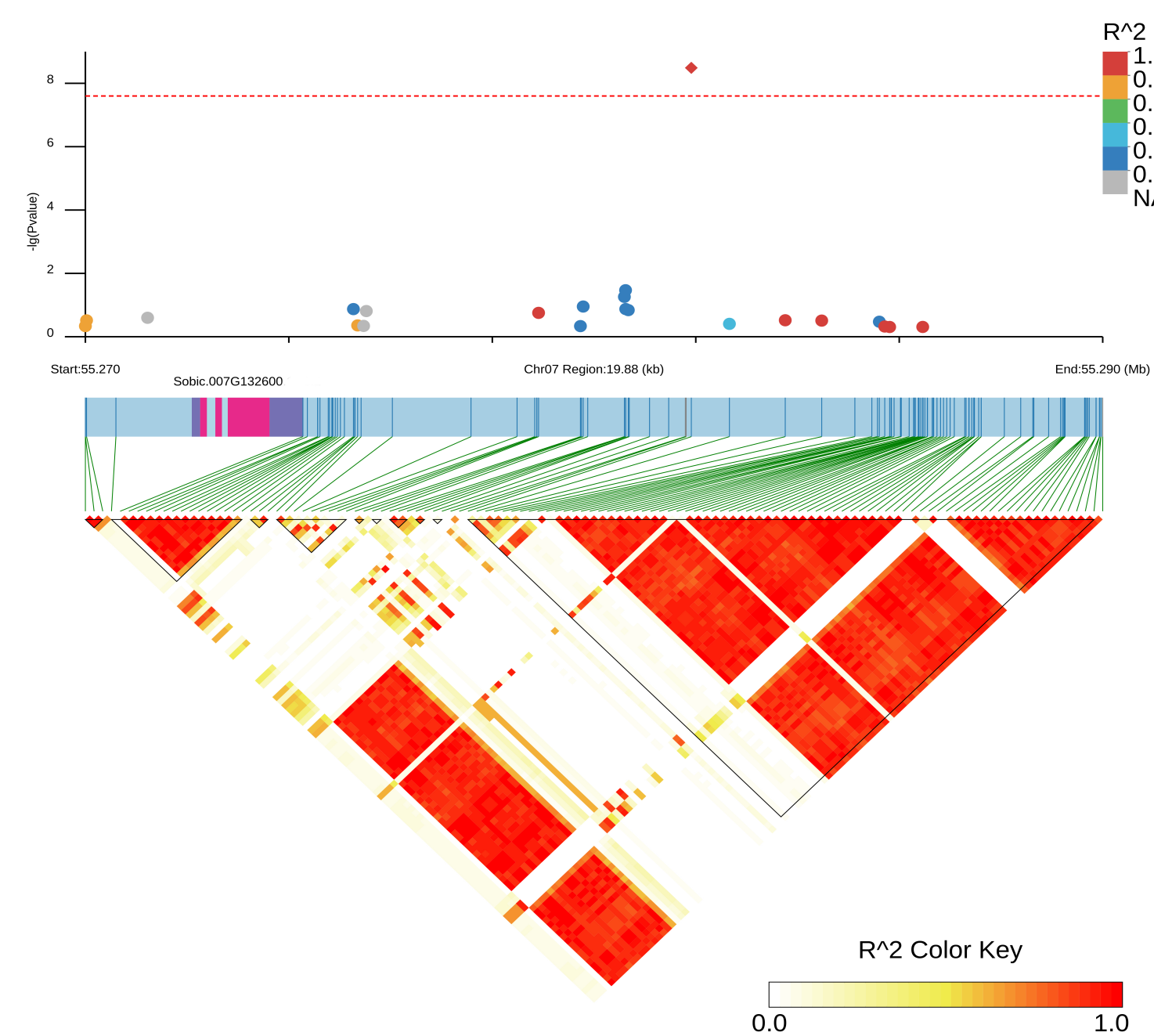

Supplement: Supplementary file 6 — Local Manhattan plot and LD heat map for important loci related to the color of exocarp, mesocarp and testa, as well as tannin content (PDF 47184 KB) [file 122_2023_4307_MOESM6_ESM.pdf]
